# Real‐Time In Vivo Monitoring of Cholinergic Neurotransmission in the Mouse Brain Using a Microelectrochemical Choline Biosensor

**DOI:** 10.1111/ejn.70291

**Published:** 2025-11-03

**Authors:** Seán Doyle, Michelle M. Doran, Colm Cunningham, John Patrick Lowry

**Affiliations:** ^1^ Neurochemistry Laboratory, Department of Chemistry Maynooth University Maynooth Co. Kildare Ireland; ^2^ School of Biochemistry and Immunology, Trinity Biomedical Sciences Institute & Trinity College Institute of Neuroscience Trinity College Dublin Dublin Ireland

**Keywords:** acetylcholine, choline, chronic recording, neurochemistry

## Abstract

The measurement of choline as a biomarker for in vivo cholinergic neurotransmission is a valuable tool in the study of a range of CNS pathologies. However, the continuous detection of cholinergic neurotransmission in selective brain regions in the mouse brain remains challenging and underexploited. Here, we have refined an established choline oxidase (ChOx) microelectrochemical biosensor and validated its use for long‐term recording in the freely moving mouse. Using a 75‐μm diameter polymer‐ChOx composite disc electrode, we have successfully monitored stable and reproducible chronic real‐time changes in choline‐induced amperometric currents in vivo. Local infusions of choline and acetylcholine resulted in an increase in biosensor current in the hippocampus, while the inhibition of endogenous acetylcholinesterase (with neostigmine) significantly attenuated the response to exogenous acetylcholine. Systemic administration of donepezil produced a pronounced decrease in current in both the prefrontal cortex and hippocampus, with scopolamine and amphetamine resulting in signal increases that were not observed in animals with selective saporin lesioning (murine‐p75) of the cholinergic basal forebrain. Furthermore, continuous biosensor recording in both regions displayed diurnal oscillations across repetitive light–dark phases. All are consistent with successful monitoring of endogenous changes in cholinergic neurotransmission.

## Graphical abstract

The measurement of choline as a biomarker for in vivo cholinergic neurotransmission is a valuable tool in the study of a range of CNS disorders. However, the continuous detection of cholinergic neurotransmission in selective brain regions in the mouse brain remains challenging and underexploited. Here, an enzyme‐modified microelectrode biosensor designed for long‐term real‐time choline monitoring in the brain has been successfully refined by changing the geometric shape of the biosensor's active surface to a disc and by miniaturising to 75 μm.

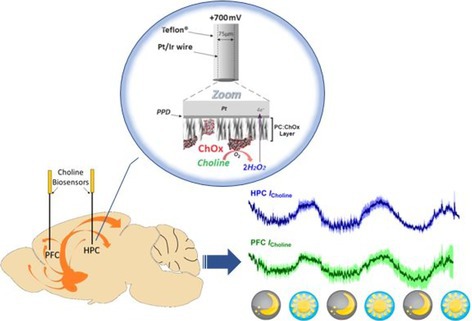

**Abbreviations**
AA: l‐ascorbic acid
AAM: Animal Activity Monitoring system (Raturn)
aCSF: artificial cerebrospinal fluid
ANOVA: analysis of variance
AUC: area under the curve
BF: basal forebrain
BSA: bovine serum albumin
ChOx: choline oxidase
CNS: central nervous system
CPA: constant potential amperometry
dHPC: dorsal hippocampus
icv: intracerebroventricular
ip: intraperitoneal injection
LPS: lipopolysaccharide
LRS: linear region slope
MMA: methyl methacrylate
mPFC: medial prefrontal cortex
*o*‐PD: *o*‐phenylenediamine
p75‐sap: p75‐Saporin neurotoxin
PBS: phosphate‐buffered saline
PC: polymer composite
PEI: polyethyleneimine
PPD: poly‐*o*‐phenylenediamine
Pt/Ir: platinum/iridium
SCE: saturated calomel electrode
SEM: standard error of the mean
ZT: Zeitgeber Time scale

## 1 Introduction

Cholinergic neurotransmission is ubiquitous throughout the central nervous system (CNS), capable of altering neuronal excitability, coordinating neuronal firing patterns and regulating neurotransmitter release (Goutier et al. 2016; Kawai et al. 2007). Thus, acetylcholine (ACh) acts as a key neuromodulator of a variety of physiological functions within the CNS, including neurovascular coupling, REM sleep, inflammation, arousal, learning and memory (De Simone et al. 2005; Lecrux et al. 2017; Papouin et al. 2017; Teles‐Grilo Ruivo et al. 2017). Cholinergic hypofunction (*hypocholinergia*) has been described in a number of degenerative disorders, including Alzheimer's disease, Parkinson's disease and delirium (Field et al. 2012; Hampel et al. 2018; Perez‐Lloret and Barrantes 2016). Furthermore, there is increasing evidence that abnormalities in cholinergic signalling contribute to a number of neuropsychiatric disorders, including schizophrenia and depression (Adams and Stevens 2007; Higley and Picciotto 2014; Mineur et al. 2013).

Cholinergic neurons arising in the basal forebrain (BF) serve as a major source of projections to cortical and subcortical structures involved in cognitive processing (Woolf 1991). Cellular subdivision within the BF topographically segregates cholinergic neurons based on their afferent and efferent projection patterns (Gielow and Zaborszky 2017; Lean et al. 2019). While ACh synthesis, receptor activation and hydrolysis are highly coordinated and well‐documented processes, the temporal and spatial resolution of cholinergic transmission remains somewhat controversial, with conflicting theories focused on the dichotomy of phasic (fast) and tonic (slow) transmission (Disney and Higley 2020; Sarter and Lustig 2020). Conventional analytical techniques, like microdialysis, have provided significant insights into the dynamics of cholinergic neurotransmission during certain cognitive processes and behavioural responses (Arnold et al. 2002; Fadel 2011; Pepeu and Giovannini 2004). However, the limited spatial and temporal resolution of these traditional techniques has led to the belief that cholinergic neurotransmission operates on a relatively slow, diffuse level. While there is strong evidence to support a correlation between alterations in tonic ACh changes and brain arousal states (Teles‐Grilo Ruivo et al. 2017; Xu et al. 2015), the use of electrochemical methods, involving amperometric microelectrode biosensors, has provided evidence of phasic bursts in cholinergic activity, consistent with transient‐synaptic release (Howe et al. 2017; Parikh et al. 2007). Electrochemical microelectrodes possess significant advantages over microdialysis, including a dramatically enhanced temporal resolution, facilitating the measurement of transmitter release on a subsecond timescale over a range of behavioural states. Additionally, microelectrodes have a smaller probe size, allowing the selective targeting of smaller brain structures.

**FIGURE 1:**
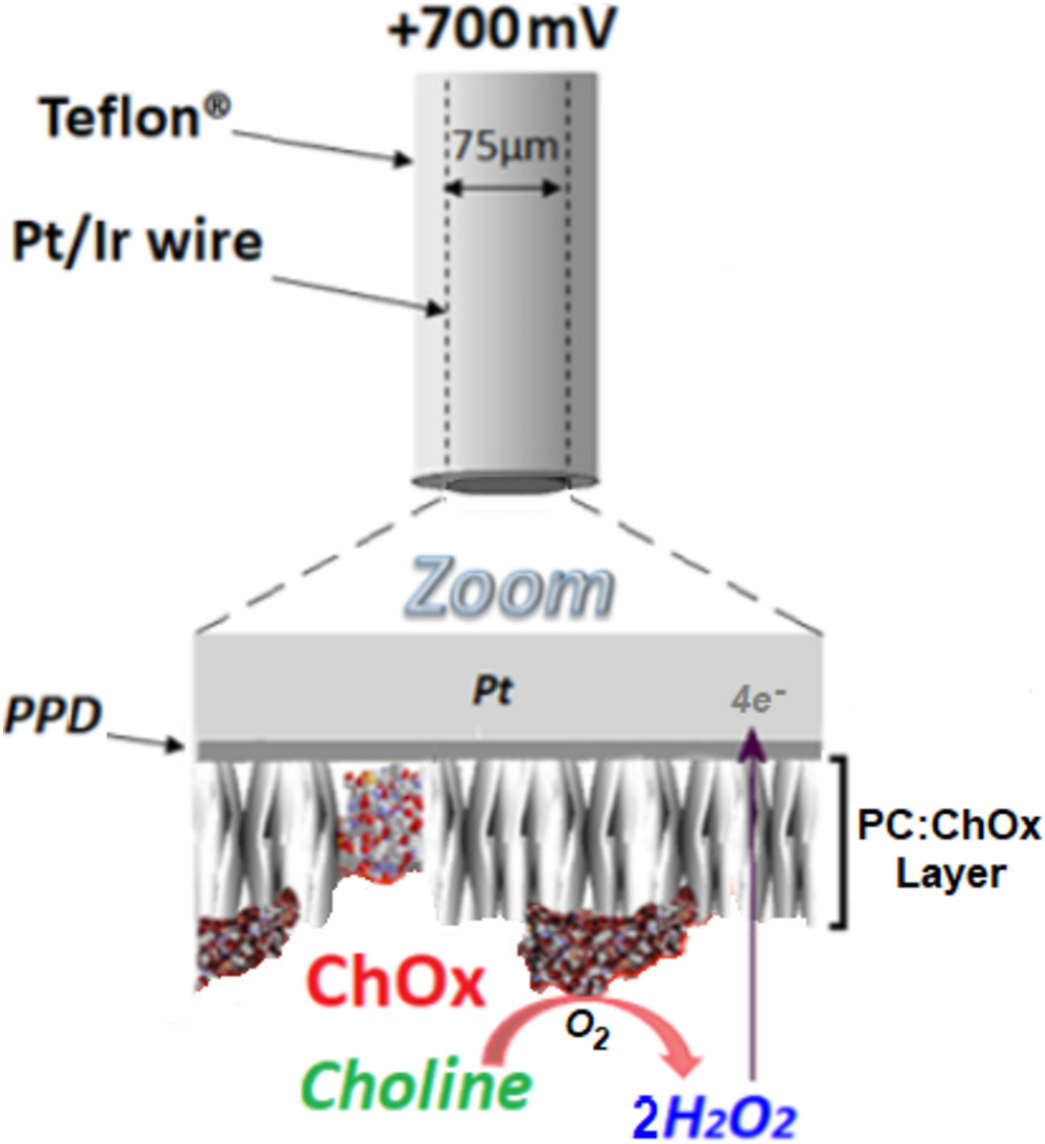
Illustration depicting a choline disc biosensor. In brief, the disc surface is coated with a layer of choline oxidase (ChOx), which oxidises choline at the electrode surface, generating the electroactive species, H_2_O_2_. The generated H_2_O_2_ is subsequently detected by a polarised (+700 mV) platinum/iridium (Pt/Ir—90/10) wire. Note the layer of electropolymerized PPD that rejects potential electroactive interferants and the polymer composite (PC) layering that immobilises and stabilises the ChOx.

In recent years, a number of choline‐selective microelectrochemical biosensors have been developed and successfully used as a valid measure of cholinergic neurotransmission in vitro (Xin and Wightman 1997a, 1997b) and in vivo (Baker et al. 2015, 2019; Mitchell 2004; Parikh et al. 2004). Using choline oxidase‐based electrochemical biosensors, nonelectroactive choline is converted to an electroactive species, H_2_O_2_, which is subsequently detected at a polarised (+700 mV) platinum (Pt) wire. In this study, we have refined the spatial resolution of a choline biosensor by altering the geometric shape of the biosensor's active surface from a cylinder to a disc and by reducing the Pt wire diameter from 125 to 75 μm (see Figure 1). While this offers clear advantages in terms of reduced tissue damage and the potential of applications in small brain regions, these could be offset by significantly poorer selectivity directly associated with the miniaturisation (McMahon et al. 2004). As such, in vitro response characteristics for both geometries were compared, and in vivo recording of cholinergic neurotransmission was further validated in the freely moving mouse.

## 2 Materials and Methods

### 2.1 Subjects and Housing

Three‐ to six‐month old female C57BL/6J mice were supplied by Charles River (Margate, UK). All animals were housed in groups of three to five siblings, maintained in a temperature (20°C–23°C) and humidity (40%–70%) controlled facility at Maynooth University, housed under a normal 12:12‐h light–dark cycle (lights on from 07:00 to 19:00) and had access to water and food ad libitum. Prior to surgery, mice were habituated to handling and group‐housed untethered in a Raturn recording bowl (BASi, West Lafayette, IN, USA). For 3–4 h/day, they were single‐housed, collared and tethered to a Raturn Animal Activity Monitoring system (AAM) (see below). During biosensor recording, mice were single‐housed for a maximum of three consecutive days per week up to a maximum of 3 weeks. All animal work was carried out with approval from Maynooth University Research Ethics Committee (BSRESC‐2017‐002) and under licence (HPRA AE19136/P065) in accordance with Part 5 of the European Union (Protection of Animals Used for Scientific Purposes) Regulations 2012 (S.I. No. 543 of 2012).

### 2.2 Chemicals and Solutions

The following were obtained from Sigma‐Aldrich Ireland Ltd. (Dublin): NaCl (SigmaUltra), NaH_2_PO_4_ (Sigma, A.C.S. reagent), KCl (SigmaUltra), CaCl_2_ (SigmaUltra), MgCl_2_ (SigmaUltra), NaHCO_3_ (99.7%, ACS reagent), d‐glucose (ACS reagent), choline chloride (> 97%), l‐ascorbic acid (AA; sodium salt, ACS reagent), bovine serum albumin (BSA; fraction V from bovine plasma), glutaraldehyde (Grade 1, 25%), *o*‐phenylenediamine (*o*‐PD; 1,2‐diaminobenzene, > 98%), polyethyleneimine (PEI; 80% ethoxylated), cellulose acetate (Mn ∼ 50,000 g/mol), methyl methacrylate (MMA; 99%), choline oxidase (from *Alcaligenes* sp., EC 232‐840‐0, 1 KU), lipopolysaccharide (LPS, from *Salmonella equine abortus*, L5886) and phosphate‐buffered saline (PBS) tablets (yield 0.01 M phosphate buffer, 0.0027 M KCl and 0.137 M NaCl when dissolved in 200‐mL deionised water, pH 7.4, at 25°C). d‐Amphetamine sulphate (> 99%), scopolamine hydrobromide (> 99%) and donepezil hydrochloride (> 99%) were obtained from Bio‐Techne Ltd. (Tocris Bioscience, UK). p75‐saporin neurotoxin (p75‐sap; mu p75‐SAP [IT‐16]) was purchased from Advanced Targeting Systems Inc. (USA).

Solutions of choline chloride (0.1 M), *o*‐PD (300 mM in N_2_‐saturated PBS), BSA (1%), cellulose acetate (2% in 2:1 acetone:ethanol), glutaraldehyde (0.5%), PEI (2%), PBS (0.01 M) and ChOx (500 U/mL in PBS) were freshly prepared as needed using Milli‐Q water (18.2 MΩ.cm). Solutions of p75‐sap were prepared in sterile PBS. All solutions for systemic administrations were prepared in sterile saline (0.9%) and given via intraperitoneal (ip) injection. All solutions for local injection/perfusion experiments were prepared in artificial cerebrospinal fluid (aCSF; pH 7.4, composition [in mM]: 128 NaCl, 3 KCl, 1 MgCl_2_, 21 NaHCO_3_, 1.3 NaH_2_PO_4_, 1 d‐glucose and 1.3 CaCl_2_) (Baker et al. 2015). aCSF osmolarity was adjusted accordingly (NaCl replacement).

### 2.3 Choline Biosensor Preparation

Changes in extracellular choline were monitored in freely moving mice using constant potential amperometry (CPA) with refined choline microelectrochemical biosensors (Baker et al. 2015; Teles‐Grilo Ruivo et al. 2017). These were constructed from Teflon‐coated, Pt/Ir (90%/10%) wire (75‐μm diameter; Advent Research Materials, UK). One end was stripped of Teflon insulation and soldered into a gold‐plated pogo pin (PlasticsOne, USA/Bilaney Consultants Ltd., UK). A fresh disc was cut at the opposite end which acted as the active surface. This surface was coated with a layer of electropolymerized poly‐*o*‐phenylenediamine (PPD; ≥ 98%), a well‐characterised interference rejection layer making the sensor highly selective for the target analyte (Baker et al. 2017, 2019; Lowry et al. 1998). In brief, a fresh deoxygenated 300‐mM solution of the monomer was prepared by dissolving 0.324 g of *o*‐PD in 10 mL of N_2_‐saturated (3 h minimum) PBS (pH 7.4). This solution was then placed in a sonic bath and continuously bubbled with N_2_ while being agitated for 10 min to ensure maximum dissolution before being transferred to a three‐electrode electrochemical cell where electrooxidative polymerisation of the active electrode surface (four biosensors/working electrodes) was carried out amperometrically for 30 min at +700 mV versus a saturated calomel reference electrode (SCE) and bare Pt wire auxiliary electrode. As *o*‐PD is easily oxidised in air, the electrochemical cell was fitted with a PTFE lid (containing custom drilled electrode and degassing holes), and a N_2_ atmosphere was maintained over the solution throughout the polymerisation process. Immediately after polymerisation, the working electrodes were removed and rinsed in Milli‐Q water. They were then allowed to dry at room temperature for at least 3 h before commencement of enzyme immobilisation/layering. For this, the PPD‐modified electrodes were initially dipped in MMA and cellulose acetate solutions and then sequentially dipped into ChOx, BSA, glutaraldehyde and PEI, using a dip‐absorption method (Baker et al. 2015; O'Neill et al. 2008). This latter process was repeated a total of 10 times, allowing a 4‐min drying period between layers, producing a Pt/PPD‐polymer composite (PC)/ChOx‐modified (Pt/PPD‐PC:ChOx) electrode. The sensors were dried for a minimum of 1 h at room temperature and stored at 4°C before use. Null electrodes were prepared using an identical protocol with the exception of the ChOx enzyme. Details on how to prepare cylinder electrodes can be found in earlier publications (Baker et al. 2019).

All choline disc biosensors were calibrated in vitro in a standard three‐electrode glass electrochemical cell in 20‐mL PBS (pH 7.4) prior to surgical implantation to ensure sensors were responsive to varying concentrations of choline. An SCE acted as the reference electrode and a bare Pt wire served as the auxiliary electrode. CPA (+700 mV) was performed in all electrochemical calibration experiments, using custom‐designed, low‐noise potentiostats (Biostat IV, ACM Instruments, UK) with a notebook PC, a PowerLab interface system (ADInstruments Ltd., UK) and LabChart (v8, ADInstruments Ltd.).

Biosensors were allowed to settle under the influence of the applied potential until the nonfaradaic current reached a stable baseline. Choline concentration was sequentially increased from 0 to 3 mM by adding aliquots of choline chloride, followed by a brief (~20 s) stirring after each aliquot. The lower limit of detection of the choline biosensors was 100 nM. Sensors were selected for implantation if the calibration current values were similar to the expected average. Additionally, AA calibrations (0–1000 μM) were performed in the same manner to confirm interference rejection at physiological levels. Sensors that responded to AA were excluded from all experiments. Pt cylinder biosensors (1 mm × 125‐μm diameter) for in vitro comparison studies were prepared as previously reported (Baker et al. 2019). H_2_O_2_ calibrations were performed as above and over the same concentration range as AA.

### 2.4 Surgery, Intracerebroventricular (icv) Injections and Choline Biosensor Implantation

Mice were anaesthetised using the volatile anaesthetic isoflurane (4% at 450 mL/min in air for induction, 0.9%–2.5% at 250 mL/min in air for maintenance, Isoflurin) using a Univentor 400 Anaesthetic Unit (AgnTho's AB, Sweden). Once surgical anaesthesia was established, the upper head was shaved, mice were positioned in a stereotaxic frame (Kopf Digital/Lazy Susan Unit, AgnTho's AB), administered a subcutaneous (sc) injection of buprenorphine (Buprecare, 0.05 mg/kg), an sc injection of lidocaine along the upper surface of the head and connected to a pulse oximeter (MouseSTAT Jr., Kent Scientific, USA). Animal body temperature was continuously recorded and maintained at 37°C throughout the surgery with a heating pad (AgnTho's AB). Under sterile conditions, the skull was exposed and cleared of overlying periosteum. The head was levelled between bregma and lambda, and craniotomies were drilled using a 0.7‐mm steel burr (Fine Science Tools GmbH, Germany) for icv injections (AP −0.22, ML ± 1.0, DV 1.9) and for guide cannula and/or biosensor implantation in the dorsal hippocampus (dHPC; AP −2.2, ML + 1.8, DV −1.75) and medial prefrontal cortex (mPFC; AP + 1.95, ML −0.30, DV −1.90). All coordinates are in mm: AP/ML coordinates with respect to bregma and DV coordinates from the surface of the brain. Additional craniotomies were drilled for a reference electrode and three support screws (BASi), one of which was wrapped with the auxiliary electrode.

In selected mice, two 1‐μL icv injections of either sterile PBS (control animals) or p75‐sap (at a concentration of 0.6 μg/μL [0.6 μg bilaterally]) were made into the lateral ventricles using a NanoFil syringe (World Precision Instruments, UK) under the control of an infusion pump (Legato 130, AgnTho's) at a rate of 0.2 nL/min. Following injection, the needle tip was left in place for 8 min to minimise reflux.

Under stereotaxic guidance, the choline biosensors and reference electrode were slowly implanted and fixed in place using dental acrylate (Dentalon, Heraeus‐Kulzer, GmbH/AgnTho's AB). The electrode pogo pins were inserted into a Delrin 12‐channel pedestal (0.3 g, PlasticsOne/Bilaney Consultants Ltd.), which was subsequently secured to the skull using dental acrylate. Postsurgery, mice were administered an sc injection of sterile saline, allowed to recover in a thermostatically controlled cage (Datesand Ltd., UK) and administered a further two Buprecare (0.05 mg/kg) ip injections within the initial 24‐h postsurgery. Animals were assessed daily for good health and allowed a minimum of 3 days before being connected to instrumentation for in vivo recording.

### 2.5 In Vivo Biosensor Recording in Freely Moving Mice

Mice were singly housed in motion‐controlled Raturn sampling cage systems (BASi). The head‐mounted Delrin pedestal (headpiece, *~*1.1 g) was connected to the potentiostat (Electrochemical and Medical Systems [EMS], UK) using a custom‐designed lightweight, flexible six‐core cable (PlasticsOne/Bilaney Consultants Ltd.). This set‐up allowed free movement of the animals during biosensor recording. Following application of the applied potential (+700 mV), mice were allowed a further 20+ h of unperturbed recording before commencing experiments. All amperometric recordings from each working electrode (biosensor) channel were recorded at 1 kHz, and a PowerLab interface system was used for analogue/digital conversion before the data were collected on a Mac computer (iMAC) running LabChart. Animal locomotor activity (movement) was simultaneously monitored using a Raturn AAM (BASi), which detected the frequency and duration of clockwise, anticlockwise and rearing activity. All pharmacological treatments were administered via ip injection with a minimum of 48 h between treatments. Treatments included sterile saline (0.9%, NaCl), donepezil (3 mg/kg), scopolamine (1 mg/kg) and amphetamine (4 mg/kg) and were administered at a volume of 10 mL/kg. All mice were weighed daily, and respective drug doses were prepared 1 h prior to injection and administered at room temperature.

### 2.6 Local Administration

Prior to sensor implantation, a choline biosensor and null electrode were carefully glued equidistant alongside a guide cannula (extending 1 mm beyond the end of the guide) and calibrated as such. All local injection/perfusion experiments were performed under acute conditions on fully anaesthetised mice.

The sensor‐guide assembly was slowly implanted into the HPC, and an infusion cannula or microdialysis probe (1 mm) was carefully inserted into the guide. Electrodes (reference/auxiliary) and the sensor were connected to the instrumentation (eDAQ QuadStat [EA164] and e‐Corder interface system [Green‐Leaf Scientific, Dublin, Ireland]), and the signal (recorded using eDAQ Chart, v5.5.23) was allowed to reach a stable baseline over a period of 3–5 h before the commencement of local administration of test solutions.

Pulse injections of fixed volumes were performed using an infusion pump (Legato 130, AgnTho's) at a rate of 80 nL/s. Microdialysis probes were continually perfused (at a rate of 2 μL/min, controlled via a Univentor 801 syringe pump [AgnTho's AB]) with aCSF or test solutions (prepared in aCSF) throughout the duration of the experiment. A UniSwitch syringe selector (BASi) was used to switch between solutions. A wash‐out period of 10 min in control aCSF was allowed before switching between solutions.

### 2.7 Determination of Basal Extracellular Concentrations

Basal extracellular choline levels were determined using non–ChOx‐coated (null) electrodes by subtracting the in vivo background current exhibited by these channels from ChOx‐coated biosensors and dividing the difference by the sensitivity (calibration slope) of the enzyme‐coated biosensor. The valid determination of the basal levels required the use of matched biosensors/electrodes, that is, displaying identical background currents (prior to analyte addition during calibration) and permselective (interference rejection) characteristics (tested using AA, see above). Where analyte concentrations from microdialysis experiments were reported as absolute values, the data were corrected for recovery using parameters (probe length/flow rate/recovery) from the literature (Helmschrodt et al. 2020; Menacherry et al. 1992; Zhao et al. 1995).

### 2.8 Histology

At the conclusion of each experiment, animals were euthanised with pentobarbital (Euthanimal, 800 mg/kg), decapitated, and the head‐pedestal was carefully removed. The brain was extracted and stored in 10% buffered formalin solution at 4°C until histological processing. Serial 50‐μm coronal sections, including the mPFC and dHPC regions, were sectioned using a vibratome (World Precision Instruments). Sections were mounted on microscope slides, stained using cresyl violet and sensor placement was examined under a light microscope.

### 2.9 Data Analysis

The linear range for in vitro calibrations was defined by *K*_M_/2 (Ford et al. 2016; O'Neill et al. 2008), and sensitivity (linear region slope [LRS]) was determined using linear regression analysis. The LOD was determined as three times the standard deviation of the 10‐μM choline signal. The selectivity coefficient, *S*% (O'Neill et al. 2008), was used to compare the permselective properties of sensors with different geometries: *S*% = 100 * *I*(AA)/*I*(H_2_O_2_) for equimolar concentrations (1 mM) of both species. For ideally selective polymer‐modified electrodes that block AA efficiently and yet allow H_2_O_2_ access to the Pt surface, *S*% approaches zero. *I*(AA) for biosensors was determined from Δ*I* (the concentration change from 0.5 to 1 mM), which is the best indicator of the interference blocking ability of the polymer (Craig and O'Neill 2003). For bare Pt electrodes *I*_lim_ was used.

Following recordings, in vivo amperometry data were low‐pass filtered at 1 Hz to minimise noise levels. All recorded electrochemical data was preliminarily processed in Microsoft Excel for Windows. The data were subsequently exported to GraphPad Prism (v10.0.2) for plotting of graphs and statistical analysis. All data are presented as mean ± standard error of the mean (SEM), with choline biosensor current given as *I*_Choline_, or as a normalised change (Δ*I*). Changes in extracellular levels were quantified as maximum increase or decrease in signal and/or area under the curve (AUC). Paired or unpaired *t* tests (two‐tailed), and one‐way ANOVA with Dunnett's post hoc analysis (unless otherwise stated), were used as appropriate. Statistical significance for all analysis was defined as *p* < 0.05.

## 3 Results and Discussion

### 3.1 In Vitro Response

Following the sensor development phase, it is important that sensor–environment interactions are characterised prior to use in the target environment (Phillips and Wightman 2003; Hu et al. 2021; Da et al. 2023). This is particularly important for neurochemical applications due to the complex chemical matrix of the brain that consists of surface modifying agents such as lipids and proteins, in addition to electrocatalysts such as AA, all of which can affect the performance of an implanted biosensor (Echizen and Freed 1986; O'Neill 1993). Previous extensive studies by our group have demonstrated the sensitivity, selectivity, O_2_ interference and stability/biocompatibility characteristics of 1‐mm cylinder Pt/PPD‐PC:ChOx microelectrochemical choline biosensors designed for neurochemical monitoring (Baker et al. 2015, 2017, 2019). In this study, to improve spatial resolution, the diameter of the electrode wire used was reduced from 125 to 75 μm, and the geometric shape of the active surface area was changed from a cylinder to a disc.

While this has the advantage of reducing tissue damage, it also facilitates applications in smaller brain regions and potential studies of cell layers within regions. Additionally, disc electrodes have previously been reported to have higher sensitivity and similar or less oxygen dependence compared to their cylinder counterparts (Baker et al. 2017; McMahon and O'Neill 2005). However, counter to these benefits/advantages are potential adverse effects on selectivity associated with altered formation of the permselective polymer. It is not safe to assume a validated design will scale down successfully, as one cannot presume that the sensitivity to different potential interfering species scales in the same proportion (McMahon et al. 2004).

**FIGURE 2:**
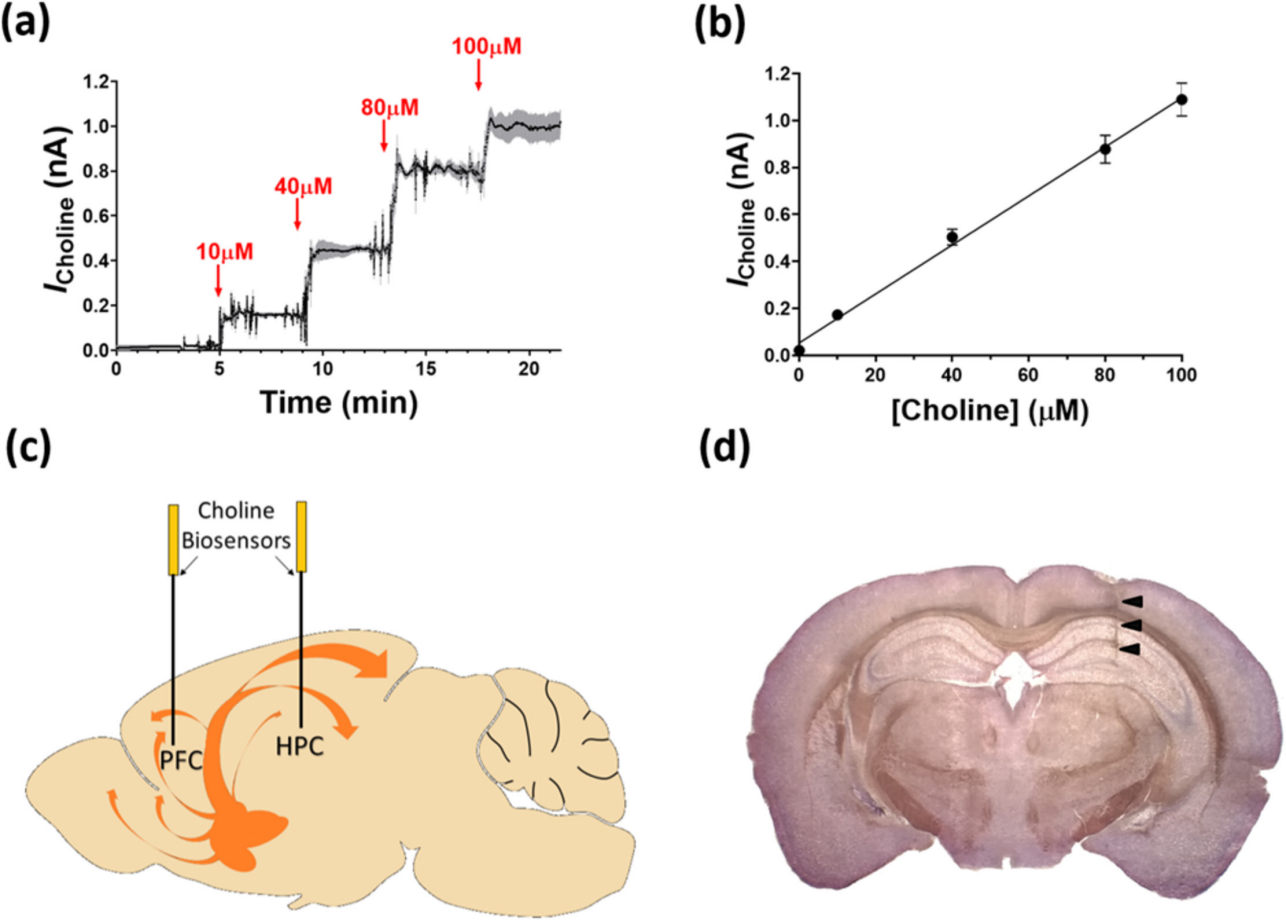
(a) Choline biosensor calibration at a range of physiologically relevant concentrations in air‐saturated PBS (pH 7.4, at +700 mV). Red arrows indicate stepwise time point of injections yielding concentrations 10, 40, 80 and 100 μM (*n* = 6, grey shadow represents SEM). Mean background current (0.021 ± 0.007 nA) subtracted. (b) Linear regression analysis of choline concentrations ranging from 0 to 100 μM (*K*_M_/2). A full calibration curve with extended liner region data is shown in Figure S1. (c) Schematic diagram of the basal forebrain cholinergic system (orange) in the mouse brain, showing the location of implanted choline biosensors in the prefrontal cortex (PFC) and hippocampus (HPC). (d) Representative image of a coronal brain slice through the hippocampus showing sensor placement (black arrows).

As such, in vitro studies were initially performed on the miniaturised choline disc biosensor to characterise sensitivity and selectivity. Calibrations were performed over a range of physiologically relevant concentrations and show a linear response at concentrations ranging from 0 to 100 μM (Figure 2a,b). The LRS was significantly increased (2.36 ± 0.15 nA/mm^2^/μM, *n* = 6; *t*_(8)_ = 5.27, *p* < 0.001, *R* ^2^ = 0.996) compared to that observed with the previous cylinder geometry (1.34 ± 0.07 nA/mm^2^/μM, *n* = 4; *R* ^2^ = 0.982) and compares favourably with carbon fibre (Garguilo and Michael 1994) (0.16 ± 0.04 nA/mm^2^/μM), Pt/Ir (Mitchell 2004) (0.90 ± 0.28 nA/mm^2^/μM) and ceramic‐based (Parikh et al. 2004) (3.74 ± 0.34 nA/mm^2^/μM) microelectrode biosensors reported by other groups.

The response characteristics for AA, generally regarded as the principal endogenous electroactive interferent (Craig and O'Neill 2003), were compared using the selectivity coefficient, *S*% (see Section 2.9). Both values were similar, close to zero (disc, 0.038 ± 0.083, *n* = 10; cylinder, 0.025 ± 0.049, *n* = 7; *t*_(15)_ = 0.123, *p* = 0.9036), and significantly reduced compared to their bare Pt counterparts (disc, 113 ± 12, *n* = 11, *t*_(12)_ = 23.09, *p* < 0.0001; cylinder, 45 ± 3, *n* = 4; *t*_(16)_ = 7.44, *p* < 0.0001), indicating that the ‘self‐sealing’ interference rejection process characteristic of PPD was not compromised by the miniaturisation of the sensor.

### 3.2 In Vivo Detection of Locally Administered Choline and Acetylcholine

Pt/PPD‐PC:ChOx disc biosensors were selectively implanted in the dHPC and mPFC, two regions that receive extensive cholinergic projections from the BF (Figure 2c) (Bloem et al. 2014; Li et al. 2018). The coronal brain section in Figure 2d depicts a representative image showing biosensor placement in the hippocampus, indicating minimal structural damage due to the small size of the electrode. This is in keeping with previous reports where the extent of tissue damage and blood–brain barrier disruption was examined following microelectrode implantation (Chatard et al. 2018; Duff and O'Neill 1994; Hascup et al. 2009) and contrasts with other conventional methods for neurochemical monitoring, such as brain microdialysis, where probes typically create a highly visible track (Clapp‐Lilly et al. 1999; Hascup et al. 2009; O'Neill et al. 1991).

**FIGURE 3:**
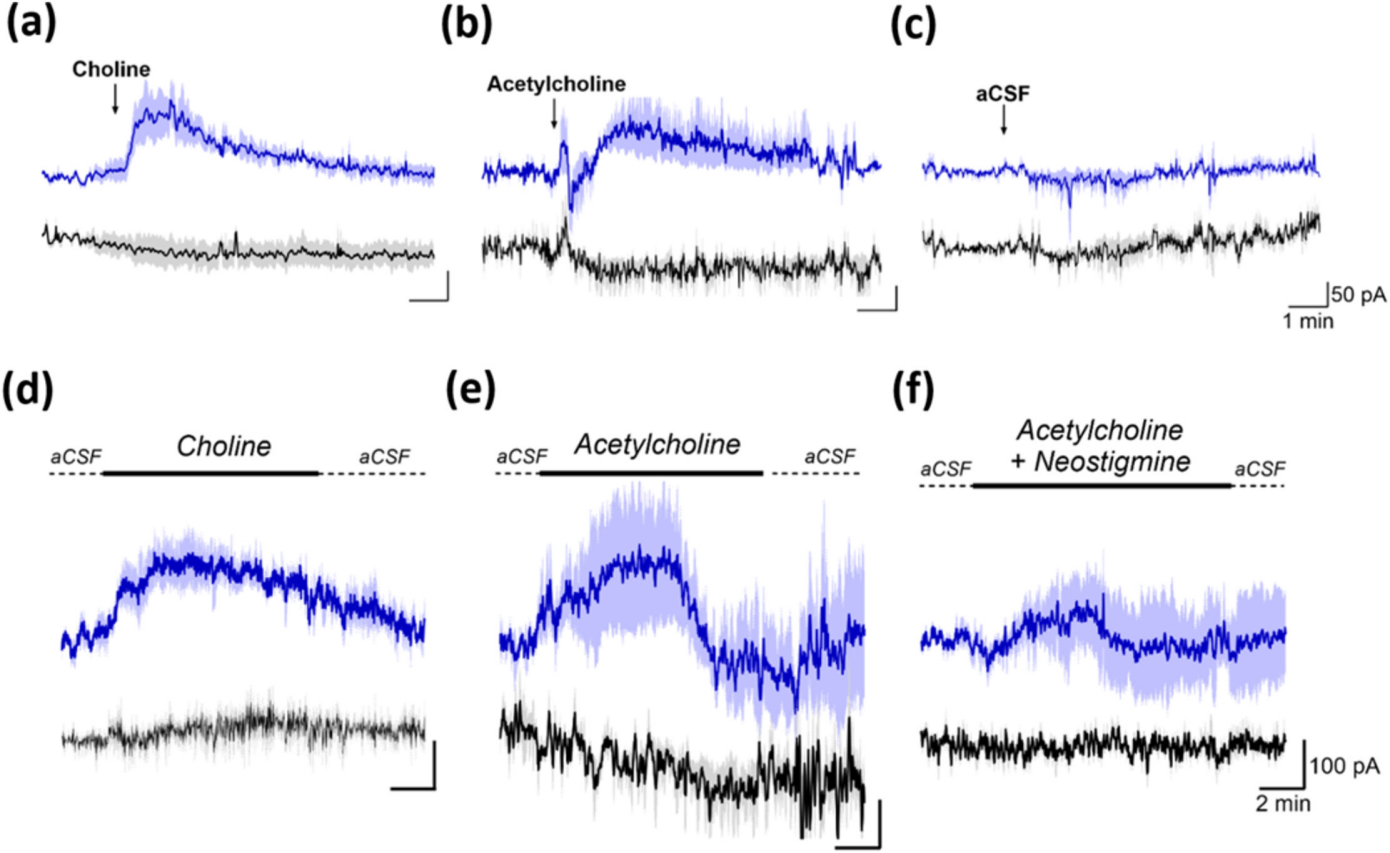
Choline biosensor (blue) and null electrode (black) responses recorded in the hippocampus following: the local pulse injection (800 nL at a rate of 80 nL/s) of choline (a, 500 mM), acetylcholine (b, 500 mM) and control aCSF (c) and the local perfusion (reverse microdialysis, at a rate of 2 μL/min) of choline (d, 500 mM), acetylcholine (e, 500 mM) and perfusate with acetylcholine (500 mM) and the acetylcholinesterase inhibitor neostigmine (100 mM) added (f). Arrows and black bars indicate the time points of injection (a–c) and periods of perfusion (d–f), respectively. Shadowing represents SEM.

Choline biosensor response in vivo was initially examined following the exogenous administration of choline and acetylcholine in anaesthetised mice. An infusion cannula, choline biosensor and null electrode were stereotaxically coimplanted in close proximity in the hippocampus. To control for non‐specific effects of the local administrations, the null electrode (devoid of ChOx) current response was monitored for each treatment. Initially, brief 10‐s pulse injections of test solutions were performed. Choline administration (500 mM, chosen to compensate for diffusion associated dilution [maximal concentration observed ~250 μM; Xin and Wightman 1997b] and to minimise the impact of uptake mechanisms) generated a pronounced increase in biosensor current (Figure 3a), increasing by a maximum of 110 ± 30 pA (*n* = 3, *t*_(4)_ = 3.439, *p* < 0.03) at 47 ± 6‐s postinjection, before gradually returning to baseline levels within 4 min.

Pulse injections of acetylcholine (500 mM, Figure 3b) resulted in a similar rise in recorded biosensor current (maximum 90 ± 30 pA, *n* = 3, *t*_(4)_ = 3.021, *p* < 0.05). However, this was slower (71 ± 6 s) compared to choline, presumably reflecting the time required for hydrolysis of acetylcholine to choline in vivo, and had only decreased by 19% after 4 min. Also noteworthy is the biphasic response that preceded the signal peak. We are unsure of the origin of this response characteristic but speculate that it may have its origins in removal (enzymatic degradation) and uptake (choline) mechanisms initially competing to address the sudden local appearance of the high acetylcholine concentration, with eventual saturation of the high‐affinity choline transporters. Administration of control aCSF produced no appreciable change in recorded choline amplitude (Figure 3c, *n* = 3): baseline signal before injection, 14.86 ± 0.63 pA; signal at 47 s, 13.67 ± 1.56 pA (*t*_(4)_ = 0.707, *p* = 0.5184); signal at 71 s, 13.69 ± 1.58 pA (*t*_(4)_ = 0.688, *p* = 0.5294). Additionally, no responses were observed at the null electrodes, confirming that the observed biosensor signal changes were caused by the injected choline and acetylcholine. These, and the observed temporal differences, are in general agreement with in vivo results from anaesthetised rats obtained with other choline and acetylcholine electrochemical biosensors (Garguilo and Michael 1996; Mitchell 2004).

Also noteworthy is the observed time delay from initiation of the microinjection to the start of the signal increase: 22 s for choline and 33 s for acetylcholine. At the injection rate used (80 nL/s), the responses would have appeared immediately if the analytes had been delivered to the biosensor by liquid flow from the injection. The observed delays imply that the bolus of fluid ejected during the injection initially remained confined to the infusion cannula and that subsequent transport of the analyte to the biosensor occurred by diffusion. As such, if we consider the small amount of exogenous analyte to be a point source of diffusing material (i.e., the radius of the injected droplet is smaller than the diffusion distance), the diffusion coefficient of the injected analyte, *D*, can be calculated by using the simplified point source expression, *D* = *d* ^2^/6*t*_max_, where *d* is the spacing between the biosensor and infusion cannula and *t*_max_ is the time required for the response to reach a maximum. Using the value of the latter given above, and a *d* value of 335 μm (determined factoring the thickness of the infusion cannula, guide cannula and Pt/Ir insulation), the calculated apparent diffusion coefficient of choline is 3.96 ± 0.60 × 10^−6^ cm^2^/s. While in reality this is an overestimate, as the point source equation assumes spherical symmetry and isotropic diffusion through the tissue, and does not allow for the effects of the finite size of the injected droplet, it is in good agreement with values previously reported for choline in brain slices (Xin and Wightman 1997b) and anaesthetised animals (Garguilo and Michael 1994) (1.8 and 2.8 × 10^−6^ cm^2^/s, respectively) and indeed those reported for a number of other small molecules in the rat brain extracellular fluid (Rice et al. 1985).

As expected, this value is less than that reported for free solution (5.8 × 10^−6^ cm^2^/s) for choline (Xin and Wightman 1997b). This decrease is mainly attributable to tortuosity (*λ*), which leads to an increased diffusion distance for small molecules due to the presence of diffusional barriers (e.g., cells) in the brain. An estimate of *λ* can be determined from the ratio (*D*_Soln_/*D*_Brain_)^1/2^, and the in vivo value obtained here (1.4 ± 0.2) is consistent with those previously reported for hippocampal slices (Nicholson 1992).

Next, choline biosensor response was examined during continuous perfusions via reverse microdialysis, which is better suited to facilitate local administration of larger volumes and drugs (Höcht et al. 2007; Mitchell 2004). Initial perfusion with aCSF resulted in a 31.6% ± 9.8% (*n* = 3) decrease in signal from the preperfusion baseline associated with the removal of analyte from the local environment of the sensor (Baker et al. 2015). Switching (UniSwitch) to choline perfusion (500 mM, Figure 3d) evoked an increase in current, reaching a maximum of 200 ± 40 pA (*n* = 4, *t*_(5)_ = 4.363, *p* < 0.01) after 2.58 ± 0.86 min, and remained elevated before gradually returning to baseline following the cessation of choline perfusion. These data are comparable to our previously observed changes for choline perfusions monitored using the larger 125‐μm cylinder biosensor in awake freely moving rats (Baker et al. 2015).

Similarly, perfusion of acetylcholine (500 mM, Figure 3e) evoked a maximum increase of 180 ± 100 pA (*n* = 4, *t*_(5)_ = 2.538, *p* < 0.06) within 2.96 ± 0.97 min. Interestingly, biosensor current returned towards baseline prior to the cessation of acetylcholine perfusion. To access the effect of acetylcholinesterase (AChE) inhibition, neostigmine (100 mM) was coperfused with acetylcholine (Figure 3f). Acetylcholine‐induced increases in biosensor current were attenuated by neostigmine, with current increasing by a maximum of 90 ± 60 pA (*n* = 4) after 2.49 ± 1.19 min. Like the acetylcholine perfusions, the recorded biosensor current returned towards baseline levels prior to switching back to control aCSF. We also repeated the pulse injection of acetylcholine (500 mM) with added neostigmine and observed attenuation of the signal increase (see Figure S2). These results, and the fact that no matching responses were observed at the null electrodes, confirm that the implanted biosensor responds to local changes in choline/acetylcholine.

By obtaining the difference between the baseline biosensor and null electrode responses, an estimate of the endogenous basal level of choline may be determined. This method has previously been applied in anaesthetised rats, with values of 4.9 (Parikh and Sarter 2006), 6.6 (Mitchell 2004) and 7.3 μM (Garguilo and Michael 1996) reported for cortex, striatum and hippocampus, respectively. Values determined in awake rats using microdialysis (6.7–13.3 [Nilsson et al. 1990] and 9.9 μM [Köppen et al. 1996] in hippocampus) are similar to basal levels determined using sensors (6.3 μM in striatum [Baker et al. 2015]). Data from mice appear to be limited to microdialysis in awake animals, where values of 7.4 (Thinnes et al. 2021) and 8.0 μM (Hartmann et al. 2008) have been reported for hypothalamus and hippocampus, respectively. The differential estimate of 12.6 ± 2.6 μM (*n* = 4) determined here using sensors in the hippocampus of anaesthetised mice is in good agreement with all of the above.

The effect of anaesthesia on extracellular acetylcholine levels has been investigated in several studies and depends on the anaesthetic, for example, injectables have been reported to decrease (pentobarbital [Damsma and Fibiger 1991; Kikuchi et al. 1997] and chloral hydrate [Bertorelli et al. 1990; Damsma and Fibiger 1991]) and increase (ketamine [Kikuchi et al. 1997]) levels, while volatiles (halothane [Damsma and Fibiger 1991] and isoflurane [Jansson et al. 2004; Shichino et al. 1997]) have been reported to suppress release. However, data for choline are unsurprisingly limited. One study reported an initial decrease followed by an increase for pentobarbital and chloral hydrate and a non‐significant decrease for halothane (Damsma and Fibiger 1991). Another study reported no change for chloral hydrate (Bertorelli et al. 1990). Isoflurane, as used here, was also found to produce non‐significant changes (Shichino et al. 1997), despite a decrease in acetylcholine, suggesting that the calculated level is a reasonable estimate.

### 3.3 Circadian Changes in Basal Choline Tone

**FIGURE 4:**
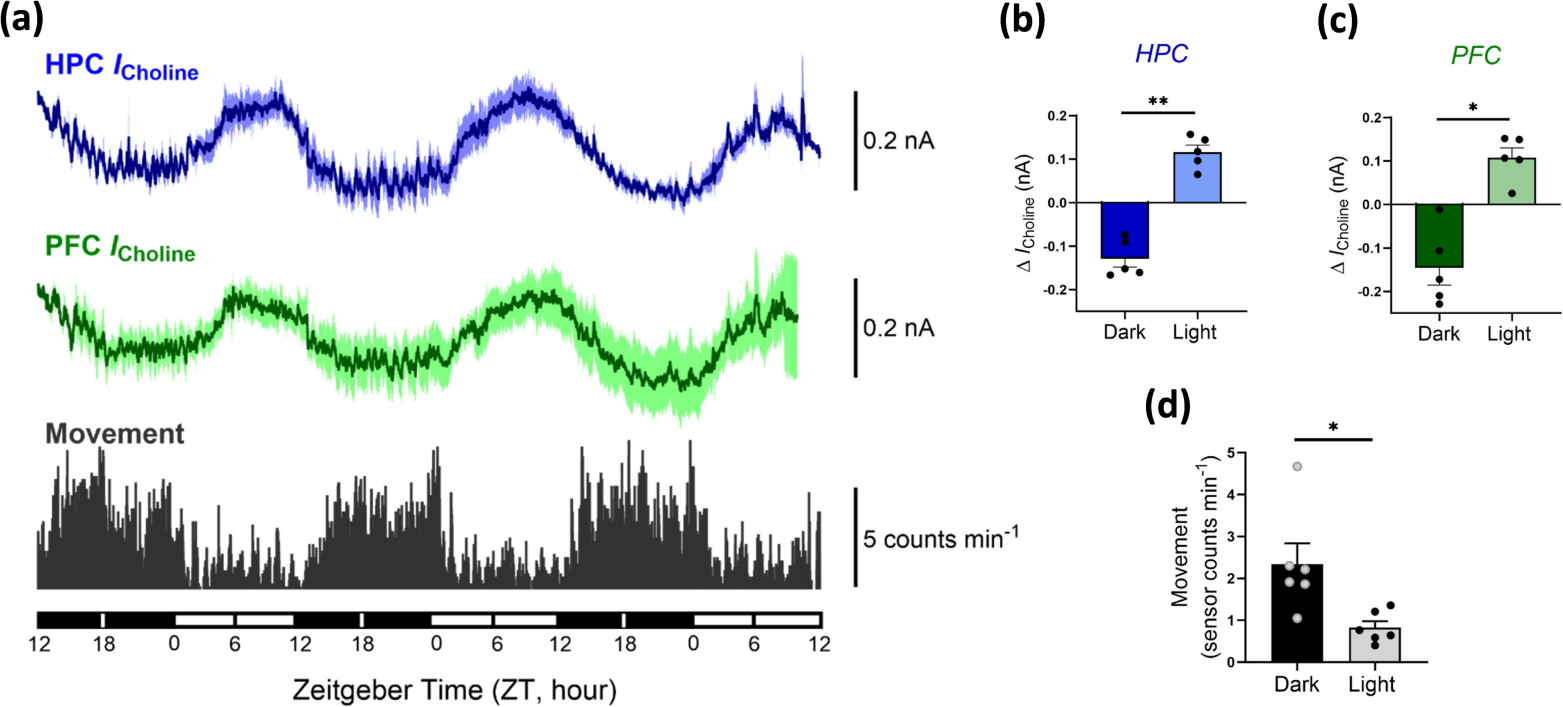
Real‐time continuous choline current recordings across three consecutive 12‐h light–dark phases in the HPC (blue) and PFC (green) of freely moving mice (a). Animal movement (grey) was simultaneously recorded for each animal (movement data are plotted in 1‐min time bins and represents the number of times the mouse moves per minute). Lighting condition is indicated by means of Zeitgeber Time scale at the bottom of the figure; open bars (ZT 0–12 h) indicate light phases, and closed bars (ZT 12–0 h) indicate dark phases. Recorded choline current was significantly higher during the light phase in both HPC (b) and PFC (c) regions. In contrast, there is a sustained increase in locomotor activity during the dark phase (d). Error bars represent SEM; *p < 0.05, **p < 0.01, paired *t* test.

Basal choline current was continuously monitored in freely‐behaving C57Bl6 mice to examine endogenous choline dynamics over consecutive light–dark phases. Previous studies have demonstrated the stability of the Pt/PPD‐PC/ChOx choline biosensors in vitro and in vivo over a period of 14 consecutive days (Baker et al. 2015, 2019). Thus, relative changes in recorded choline current will reflect endogenous fluctuations in extracellular choline levels. Continuous choline recording here revealed significant diurnal oscillations in mice across three repetitive light–dark cycles in both HPC (*t*_(4)_ = 7.65, *p* < 0.01) and PFC (*t*_(4)_ = 4.56, *p* < 0.05) regions (Figure 4a–c). Interestingly, choline levels were significantly higher during the light phase, peaking 2.70 (ZT 9.30 ± 0.46 h, *n* = 5) and 2.96 h (ZT 9.04 ± 0.40 h, *n* = 5) before the onset of the dark phase in HPC and PFC regions, respectively. Similarly, decreasing choline levels during the dark phase typically reached minimum levels at 2.44 (ZT 21.56 ± 0.61 h, *n* = 5) and 2.78 h (ZT 21.22 ± 0.69 h, *n* = 5) before light, in the HPC and PFC, respectively. To examine the relationship between locomotor activity and choline across consecutive light–dark phases, mouse movement was simultaneously recorded. As a nocturnal species, mice are predominantly active during the dark phase, spending more time asleep during the light phase. As such, daily fluctuations in locomotor activity are often used as an index of circadian rhythm (Jud et al. 2005). As expected, mouse movement was significantly higher during the dark phase (Figure 4d; *t*_(6)_ = 2.91, *p* < 0.05).

Studies on circadian rhythms in choline and acetylcholine are again predominated by the latter. A literature review revealed only two publications where choline levels were monitored (both from the early 1990s and involving in vivo microdialysis), with no clear pattern found in frontal cortex, hippocampus or striatum (Day et al. 1991; Kametani and Kawamura 1991). While early studies of changes in acetylcholine from the 1970s and 1980s involved analysis of brain homogenates and were contradictory (Friedman and Walker 1972; Hanin et al. 1970; Saito et al. 1975), more recent microdialysis studies have shown that cortical and hippocampal acetylcholine release in the rat brain follows a circadian rhythm, with dialysate acetylcholine concentrations highest during the dark phase (Hut and Van der Zee 2011; Jiménez‐Capdeville and Dykes 1993; Kametani and Kawamura 1991), correlating with locomotor activity (Dai et al. 2009; Kikuchi et al. 2013; Mizuno et al. 1991). Interestingly, endogenous acetylcholine rhythmicity appears absent in the suprachiasmatic nucleus of rats (Murakami et al. 1984), despite its well‐recognised role in regulating circadian rhythms (Hut and Van der Zee 2011).

Here, we found a clear rhythmicity for choline in the mouse brain with levels highest during the light (inactive) phase. While the circadian aspects of the cholinergic system have been reported to be variable with age and sex (Masuda et al. 2005), and apparently flexibly adjustable to the needs of a strain or species (Hut and Van der Zee 2011), cholinergic enzyme activity appears to fluctuate in a distinct pattern with higher AChE (Schiebeler and von Mayersbach 1974) and lower choline acetyltransferase (Greco et al. 1999) activity during the light phase, aligned with a reciprocal relationship of lower acetylcholine and higher choline.

It is important to remember that choline is both a precursor and degradation product of acetylcholine (Klein 2000; Klein et al. 1991) and that it has physiological functions not associated with neurotransmission (e.g., cell membrane integrity and lipid transport [Sanders and Zeisel 2007]). As such, a significant component of the basal choline signal is not due to neuronal acetylcholine release (Bruno et al. 2006; Parikh et al. 2004), and the reciprocal relationship highlighted above is not unexpected and has previously been observed following administration of certain choline transport blockers, dopamine antagonists and anaesthetics (Bruno et al. 2006; Ikarashi et al. 1997).

While earlier work has shown that microelectrode choline biosensors are a valid tool for monitoring extracellular choline as a marker for acetylcholine release (Garguilo and Michael 1996; Giuliano et al. 2008; Parikh et al. 2004; Sarter et al. 2009), one must acknowledge that while this is true under some conditions (e.g., changes associated with specific behaviours and pharmacological treatments [Bruno et al. 2006; Parikh et al. 2007; Parikh et al. 2004; Teles‐Grilo Ruivo et al. 2017]), it is unlikely to be the case for all, highlighting the need for signal validation, and ideally, where appropriate (Giuliano et al. 2008), the use of a biosensor that can monitor both analytes simultaneously (Bruno et al. 2006; Burmeister et al. 2008; Mitchell 2004).

### 3.4 Pharmacological Characterisation of Choline Biosensors

**FIGURE 5:**
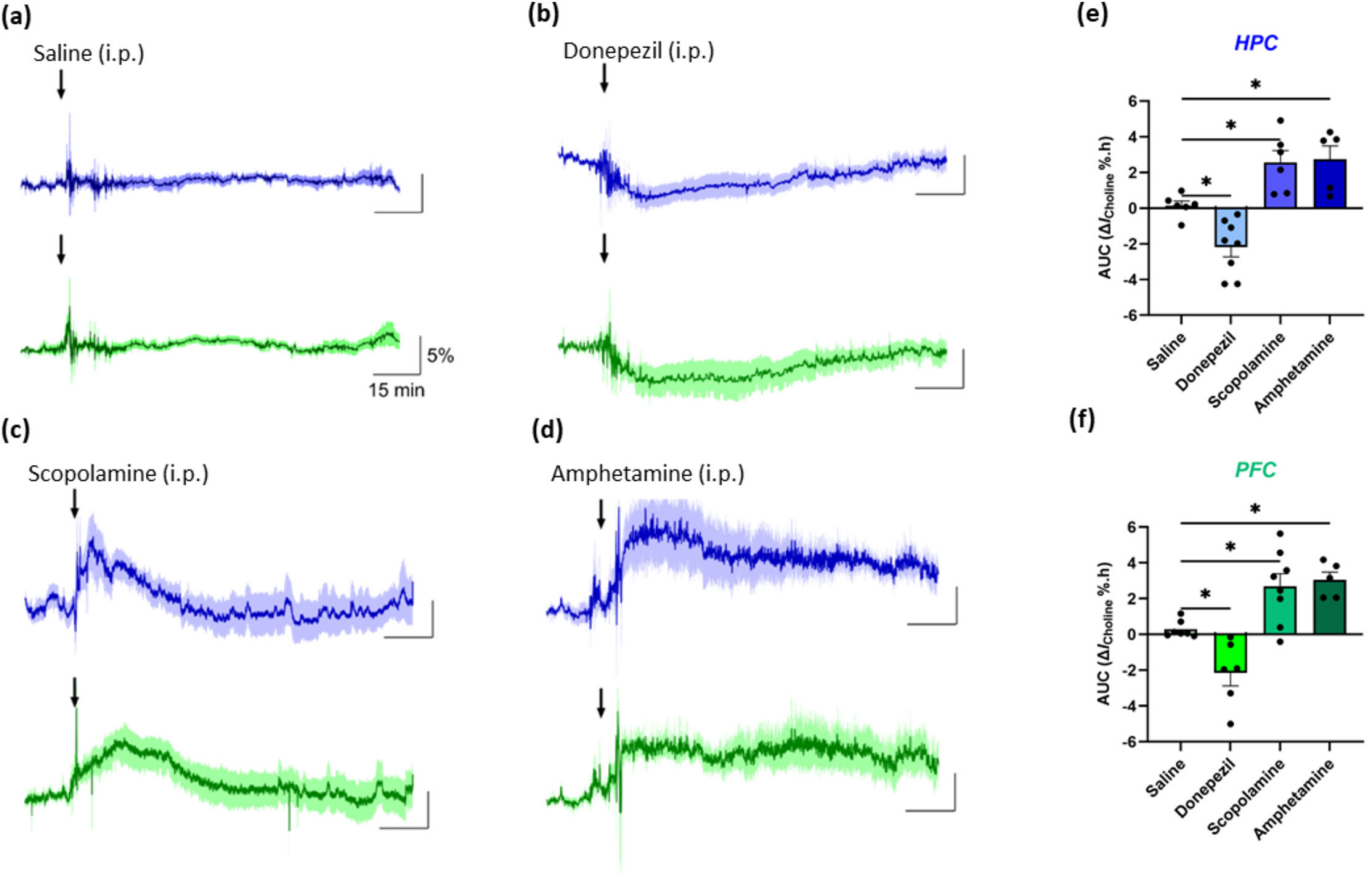
Choline current responses (mean ± SEM) in the HPC (blue) and PFC (green) following an intraperitoneal (ip) injection of sterile saline (a, 10 mL/kg), donepezil (b, 3 mg/kg), scopolamine (c, 1 mg/kg) and amphetamine (d, 4 mg/kg). Arrows indicate time points of injection. Data are normalised to a preinjection baseline and presented as relative percentage current change over time. Quantification of choline biosensor changes determined as area under the curve (AUC [Δ*I*%.h], see Table 1) following all treatments in the HPC (e) and PFC (f). Significance was determined with one‐way ANOVA and Dunnett's post hoc test, *p < 0.05.

Systemic administrations of a range of pharmacological agents known to affect central cholinergic neurotransmission were used to assess biosensor response to endogenous changes in extracellular choline levels. A preinjection baseline of 10 min was recorded before each treatment. Administration of sterile saline (0.9%, ip) typically evoked a small and rapid transient rise (HPC 1.22% ± 0.01%, *n* = 6; PFC 2.11% ± 0.15%, *n* = 7) in recorded choline current (Figure S3), which was short‐lived, with levels quickly returning to the preinjection baseline within 5 min in both HPC and PFC (Figure 5a). This is likely associated with an aversive ‘injection stress’ response (Baker et al. 2015; Lowry and Fillenz 2001) in some animals and was not previously observed with microdialysis in rats when sampling was performed at 20 (Ikarashi et al. 1997) or 15‐min (Jackson et al. 1994) intervals, although there is some evidence in data traces with 10‐min sampling (Damsma et al. 1987; Day et al. 1991).

In contrast, all pharmacological treatments produced slower, longer lasting changes in recorded choline current (Figure 5 and Table 1). Administration of donepezil (3 mg/kg, ip) produced a pronounced drop in HPC and PFC choline current, decreasing to a minimum of 6.62% ± 1.40% by 11.4 ± 2.0 min (*n* = 8, *t*_(7)_ = 4.73, *p* < 0.01) and 7.54% ± 2.41% by 8.4 ± 1.9 min (*n* = 6, *t*_(5)_ = 3.11, *p* < 0.05) postinjection, respectively (Figure 5b). In comparison, ip injection of scopolamine (1 mg/kg) induced a sharp rise in recorded choline current, increasing to a maximum of 11.80% ± 2.63% at 10.7 ± 2.9 min (*n* = 6, *t*_(5)_ = 3.87, *p* < 0.05) in the HPC and to 9.36% ± 1.18% at 19.7 ± 4.5 min (*n* = 8, *t*_(7)_ = 7.74, *p* < 0.001) in the PFC (Figure 5c). Similarly, amphetamine (4 mg/kg, ip) evoked an early and sustained rise in recorded choline current in both regions, remaining elevated for typically up to 3 h before gradually returning to baseline over a further 3–4 h. Increases of 9.32% ± 2.27% at 15.4 ± 3.2 min (*n* = 5, *t*_(4)_ = 3.74, *p* < 0.05) postinjection in the HPC and 12.90% ± 3.11% at 19.0 ± 10.3 min (*n* = 5, *t*_(4)_ = 4.18, *p* < 0.05) postinjection in the PFC were observed (Figure 5d).

**TABLE 1:** Choline biosensor responses to control and pharmacological treatments.

Treatment	Brain region	*T*_Min/Max_ (min)	Δ*I*_Choline_ (relative %)	*p*	AUC (Δ*I*%.h)	*p*
Saline	HPC	—	−0.72 ± 0.77 (6)^a^	0.3939	0.15 ± 0.26 (6)	—
	PFC	—	−0.21 ± 0.47 (7)^a^	0.6664	0.29 ± 0.18 (7)	—
Donepezil	HPC	11.4 ± 2.0	−6.62 ± 1.40 (8)	< 0.01	−2.18 ± 0.54 (8)	< 0.05
	PFC	8.4 ± 1.9	−7.54 ± 2.14 (6)	< 0.05	−2.14 ± 0.73 (6)	< 0.05
Scopolamine	HPC	10.7 ± 2.9	11.80 ± 2.63 (6)	< 0.05	2.57 ± 0.66 (6)	< 0.05
	PFC	19.7 ± 4.5	9.36 ± 1.18 (8)	< 0.001	2.68 ± 0.72 (8)	< 0.05
Amphetamine	HPC	15.4 ± 3.2	9.32 ± 2.27 (5)	< 0.05	2.74 ± 0.76 (5)	< 0.05
	PFC	19.0 ± 10.3	12.90 ± 3.11 (5)	< 0.05	3.05 ± 0.44 (5)	< 0.05

^a^ Calculated at mean *T*_Min/Max_ (14.1 min) for all pharmacological treatments. Numbers in parentheses = *n*.

The signal changes observed as a consequence of these targeted pharmacological treatments confirm the source of the measured current is extracellular choline. Donepezil is a selective and reversible AChE inhibitor that is known to increase acetylcholine levels in several brain regions (Kosasa et al. 1999; Liang and Tang 2004; Naik et al. 2009; Yamahashi et al. 2022) and has also been reported to decrease choline in both PFC and striatum (Hassani et al. 2023). In the latter, choline concentrations were reduced by *~*80% of baseline levels and were used as a metric for donepezil activity, which both deactivates AChE and also prevents acetylcholine degradation to choline. This reciprocal relationship highlights the importance of ideally striving to simultaneously monitor the two analytes in order to better understand their intimate connection, particularly given the mounting evidence that choline supplementation may benefit individuals with cognitive impairment or neurodegenerative diseases (Aguree et al. 2023; Velazquez et al. 2019).

While the measurements reported herein facilitate useful insights into the relationship through comparisons with the extensive acetylcholine literature, the real potential of simultaneous monitoring is highlighted by the small number of early microdialysis studies that measured both and reported dissociations for treatments involving anaesthesia/hypothermia, agonists/antagonists (e.g., dopamine) and choline transport blockers (Damsma and Fibiger 1991; Ikarashi et al. 1997), with results suggesting that in brain regions rich in cholinergic innervation, extracellular choline changes are primarily determined by the activity of cholinergic transmission reflected in high‐affinity choline transporter activity. In regions where cholinergic innervation is sparse, the extracellular levels may be determined predominantly by phosphatidylcholine metabolism (Löffelholz et al. 1993).

It is well documented that muscarinic antagonists such as scopolamine increase PFC and HPC acetylcholine efflux (Day et al. 1991; Jackson et al. 1994; Toide and Arima 1989). However, its effect on choline is variable, with dialysate levels showing no change in both regions (Day et al. 1991), and decreases (Jackson et al. 1994; Toide 1989). Contrary to this, real‐time increases in cortical concentrations have been reported using biosensors (microelectrode arrays) (Parikh et al. 2004) and confirmed to reflect acetylcholine arising from presynaptic muscarinic receptor blockade.

Contrasting results have also been found with the psychostimulant and indirect dopamine agonist amphetamine, with increases (Arnold et al. 2001; Hedou et al. 2000; Imperato et al. 1993; Jönsson et al. 1969; Lindefors et al. 1992; Schmidt 1976; Trabucchi et al. 1975) reported in some studies and decreases (Damsma et al. 1991; DeBoer and Abercrombie 1996; Domino and Olds 1972; Hedou et al. 2000; Schmidt 1976; Vasko et al. 1974) or no change (DeBoer and Abercrombie 1996; Herrera‐Marschitz et al. 1994) in others for both acetylcholine and choline. Again, the majority of these studies involved microdialysis, and it is likely that the variability observed may be related to the different doses used (DeBoer and Abercrombie 1996) and the added complication of the addition of AChE inhibitors to the perfusion solution in order to improve acetylcholine recovery and detection (Damsma et al. 1987; Hedou et al. 2000). It has been demonstrated that this artificial increase in basal acetylcholine levels can change the mechanisms by which both cholinergic and dopaminergic compounds affect regional acetylcholine release (Acquas and Fibiger 1998; de Boer et al. 1990).

### 3.5 Hypocholinergic Mouse Model.

**FIGURE 6:**
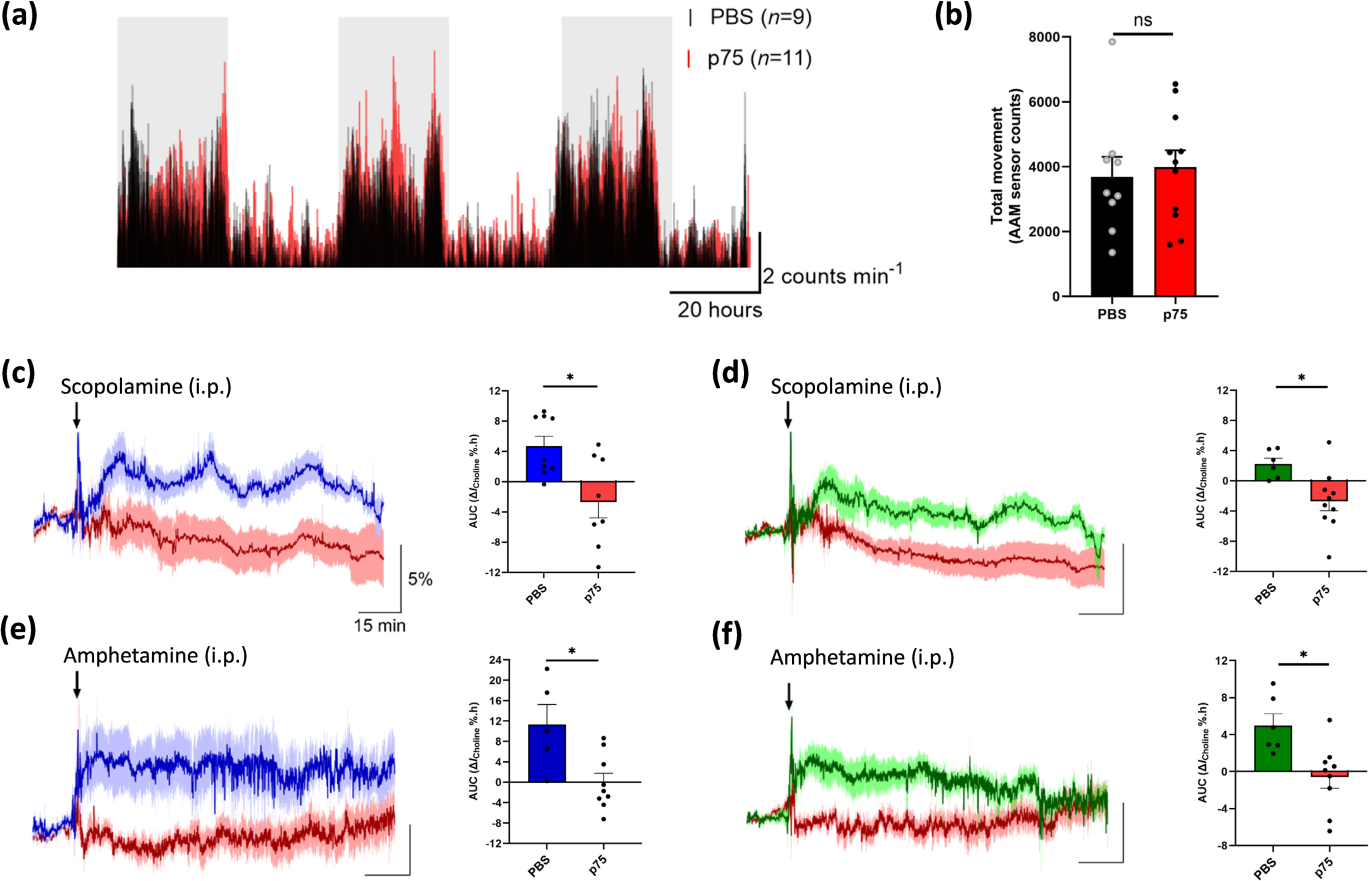
Locomotor activity monitored using a Raturn Animal Activity Monitoring system (AAM) over three consecutive 24‐h dark/light cycles in animals with selective sap lesioning of the cholinergic basal forebrain (p75, red) and sham‐lesioned (PBS, black) controls (a,b). Choline current responses (mean ± SEM) in the HPC (blue) and PFC (green) from lesioned and control animals following intraperitoneal (ip) injection of scopolamine (c,d, 1 mg/kg) and amphetamine (e,f, 4 mg/kg). Arrows indicate time points of injection. Data are normalised to preinjection baseline and presented as relative percentage current change over time. Quantification of changes was determined as area under the curve (AUC, Δ*I*%.h, see Table 2) with p75‐sap induced hypocholinergia significantly (*p < 0.05, unpaired *t* test) attenuating the pharmacologically evoked choline increases for both drugs.

Selective lesioning of the cholinergic BF is regularly used as a tool to examine the significance of losing cholinergic tone, a hallmark characteristic associated with neurodegenerative disorders like Alzheimer's disease (Field et al. 2012; Nag et al. 2009). Previous studies have produced and characterised a hypocholinergic mouse model using a ribosome inactivating immunotoxin, murine‐p75‐sap (p75‐sap), to selectively lesion cholinergic neurons in the BF (Field et al. 2012). The resultant depletion of cholinergic density was found to decrease cholinergic innervation to projected hippocampal regions (Field et al. 2012) and also to significantly lower the choline current response to systemic challenge with lipopolysaccharide in both the hippocampus and prefrontal cortex compared to PBS controls (Figure S4) (Nazmi et al. 2021). To further validate the choline biosensor in vivo, a response study was performed in selected p75‐sap (1.2 μg) lesioned mice. Lesioning had no significant effect on overall locomotor activity when compared to PBS controls (Figure 6a,b, *t*_(18)_ = 0.376, *p* = 0.7115), consistent with previous reports where no difference was observed in spontaneous locomotion between sap and sham‐lesioned controls in both rats (65 min [Mattsson et al. 2005]) and mice (36 h, incorporating two dark (wake) and one light (sleep) cycle [Hamlin et al. 2013]).

Average baseline biosensor signals measured over the 72 h in both regions were also not significantly different: HPC 1.21 ± 0.09 (PBS, *n* = 6) and 1.27 ± 0.10 nA (p75‐sap, *n* = 10), *t*_(14)_ = 0.385, *p* = 0.7064; PFC 1.88 ± 0.15 (PBS, *n* = 6) and 1.75 ± 0.09 nA (p75‐sap, *n* = 10), *t*_(14)_ = 0.792, *p* = 0.4417. This is not unexpected as basal choline (Parikh and Sarter 2006) and acetylcholine (Laursen et al. 2014; Watanabe et al. 2009) levels appear to be unaffected by sap lesioning where there is not total loss of cholinergic neurons, as is the case here (Berger‐Sweeney et al. 2001; Nag et al. 2009). Under such partial lesioning conditions, it has been proposed that sufficient levels of functional neurons remain intact such that the cholinergic system is able to retain its capacity to release neurotransmitter for basal signalling under normal conditions, with signalling only compromised during conditions of elevated neurotransmitter release (Laursen et al. 2014). In agreement with this, the rise in recorded choline current was significantly attenuated (Table 2) in the p75‐sap lesioned animals, following the administration of scopolamine (HPC: Figure 6c, *t*_(15)_ = 2.74, *p* < 0.05; PFC: Figure 6d, *t*_(14)_ = 2.83, *p* < 0.05) and amphetamine (HPC: Figure 6e, *t*_(12)_ = 2.59, *p* < 0.05; PFC: Figure 6f, *t*_(13)_ = 2.65, *p* < 0.05), consistent with decreased cholinergic innervation to both regions, and demonstrating that the observed drug‐induced increases require acetylcholine release and hydrolysis.

**TABLE 2:** Comparison of choline biosensor responses to scopolamine and amphetamine in PBS control and p75‐sap lesioned mice.

Treatment	Brain region	PBS AUC (Δ*I*%.h)	p75 AUC (Δ*I*%.h)	*p*
Scopolamine	HPC	4.76 ± 1.42 (9)	−2.56 ± 2.34 (8)	< 0.05
	PFC	2.23 ± 0.76 (6)	−2.70 ± 1.26 (10)	< 0.05
Amphetamine	HPC	11.01 ± 4.41 (5)	0.04 ± 2.06 (9)	< 0.05
	PFC	4.99 ± 1.25 (6)	−0.54 ± 1.48 (9)	< 0.05

*Note:* Numbers in parenthesis = *n*.

Interestingly, analysis of the choline signal over consecutive light–dark phases (Figure S5) revealed a similar circadian pattern to that observed for normal untreated animals, with significantly higher currents during the light phase in both HPC and PFC for both sham and p75‐sap lesioned animals. However, the currents were attenuated in the latter (HPC: 47% [dark] and 73% [light] decrease; PFC: 57% [dark] and 57% [light] decrease), most likely reflecting the loss of cholinergic terminals and consequent decrease in the number of high‐affinity choline transporters available for choline uptake (Parikh and Sarter 2006).

## 4 Conclusions

In summary, a choline oxidase‐based microelectrode biosensor designed for chronic real‐time monitoring in the brain has been successfully refined by changing the geometric shape of the biosensor's active surface from a cylinder to a disc and by reducing the diameter from 125 to 75 μm. In vitro studies confirmed the retention of interference rejection properties and a sensitivity comparable to previously reported sensors. Novel and targeted in vivo experiments in mice involving local infusions of choline and acetylcholine, systemic administration of cholinergic drugs and lesion studies, all validated successful real‐time monitoring of cholinergic neurotransmission in both the prefrontal cortex and hippocampus.

## Author Contributions

**Seán Doyle:** data curation, formal analysis, investigation, methodology, supervision, validation, visualization, writing – original draft. **Michelle M. Doran:** formal analysis, investigation, methodology, resources. **Colm Cunningham:** conceptualization, funding acquisition, supervision. **John Patrick Lowry:** conceptualization, data curation, formal analysis, funding acquisition, investigation, methodology, supervision, validation, visualization, writing – original draft, writing – review and editing.

## Conflicts of Interest

The authors declare no conflicts of interest.

## Peer Review

The peer review history for this article is available at https://www.webofscience.com/api/gateway/wos/peer‐review/10.1111/ejn.70291.

## Supporting information


**Figure S1:** (a) Choline calibration data (current vs. concentration) for Pt/PPD‐PC:ChOx disc biosensors (*n* = 6) over the range 0 to 3000 μM choline. (b) Linear regression analysis of choline concentrations ranging from 0 to 200 μM (sensitivity 2.26 ± 0.06 nA/mm^2^/μM, *R* ^2^ = 0.962; limit of detection 0.14 ± 0.04 μM).
**Figure S2:** Choline biosensor (blue) and null electrode (black) responses recorded in the hippocampus following the local pulse injection (800 nL at a rate of 80 nL/s) of acetylcholine (500 mM, a) and acetylcholine/neostigmine (500 mM/100 mM, b). Acetylcholine produced a maximum current increase of 90 ± 30 pA at 71 ± 6 s (*n* = 3). This was attenuated by the neostigmine with the current increasing by 3.4 ± 0.4 pA (*n* = 2) at the same time point following injection. No responses were observed at the null electrodes. Arrows indicate time point of injection, and shadowing represents SEM.
**Figure S3:** Choline current responses (mean ± SEM) in the HPC (blue) and PFC (green) following an intraperitoneal injection of sterile saline (10 mL/kg). Data are normalised to the preinjection baseline and presented as relative percentage current change over time. Arrows indicate time point of injection and shadowing represents SEM.
**Figure S4:** Choline current responses (mean ± SEM) in the HPC and PFC from PBS control (a,b) and p75‐sap (0.6 μg bilaterally) lesioned (c,d) mice following intraperitoneal injection (ip) of LPS (500 μg/kg; PBS—blue/green, p75—red) or sterile saline (10 mL/kg, grey). Data are normalised to the preinjection baseline and presented as relative percentage current change over time. Arrows indicate time point of injection and shadowing represents SEM.
**Figure S5:** Choline signal changes (mean ± SEM) in the HPC (a) and PFC (c) from PBS control (blue, green) and p75‐sap lesioned (red) mice across three consecutive 12‐h light–dark phases. Lighting condition is indicated by means of Zeitgeber Time scale at the bottom of the figures; open bars (ZT 0–12 h) indicate light phases, closed bars (ZT 12–0 h) indicate dark phases.

## Data Availability

The data that support the findings of this study are available from the corresponding author upon request.
