# Supplementary material for: Real‐Time In Vivo Monitoring of Cholinergic Neurotransmission in the Mouse Brain Using a Microelectrochemical Choline Biosensor
Source: Eur J Neurosci. 2025 Nov 3;62(9):e70291. doi: 10.1111/ejn.70291 (PMC12583884; doi:10.1111/ejn.70291)
Supplement: Supplementary file 1 — Figure S1: (a) Choline calibration data (current vs. concentration) for Pt/PPD‐PC:ChOx disc biosensors (n = 6) over the range 0 to 3000 μM choline. (b) Linear regression analysis of choline concentrations ranging from 0 to 200 μM (sensitivity 2.26 ± 0.06 nA/mm2/μM, R 2 = 0.962; limit of detection 0.14 ± 0.04 μM). Figure S2: Choline biosensor (blue) and null electrode (black) responses recorded in the hippocampus following the local pulse injection (800 nL at a rate of 80 nL/s) of acetylcholine (500 mM, a) and acetylcholine/neostigmine (500 mM/100 mM, b). Acetylcholine produced a maximum current increase of 90 ± 30 pA at 71 ± 6 s (n = 3). This was attenuated by the neostigmine with the current increasing by 3.4 ± 0.4 pA (n = 2) at the same time point following injection. No responses were observed at the null electrodes. Arrows indicate time point of injection, and shadowing represents SEM. Figure S3: Choline current responses (mean ± SEM) in the HPC (blue) and PFC (green) following an intraperitoneal injection of sterile saline (10 mL/kg). Data are normalised to the preinjection baseline and presented as relative percentage current change over time. Arrows indicate time point of injection and shadowing represents SEM. Figure S4: Choline current responses (mean ± SEM) in the HPC and PFC from PBS control (a,b) and p75‐sap (0.6 μg bilaterally) lesioned (c,d) mice following intraperitoneal injection (ip) of LPS (500 μg/kg; PBS—blue/green, p75—red) or sterile saline (10 mL/kg, grey). Data are normalised to the preinjection baseline and presented as relative percentage current change over time. Arrows indicate time point of injection and shadowing represents SEM. Figure S5: Choline signal changes (mean ± SEM) in the HPC (a) and PFC (c) from PBS control (blue, green) and p75‐sap lesioned (red) mice across three consecutive 12‐h light–dark phases. Lighting condition is indicated by means of Zeitgeber Time scale at the bottom of the figures; open bars (ZT 0–12 h) indicate li [file EJN-62-0-s001.docx]

**Supporting Information**

**Real-time *In Vivo* Monitoring of Cholinergic Neurotransmission in the Mouse Brain Using a Microelectrochemical Choline Biosensor**

Seán Doyle,^1^ Michelle M. Doran,^1^ Colm Cunningham,^2^ and John P. Lowry^1^

^1^Department of Chemistry, Maynooth University, Maynooth, Co. Kildare, Rep. of Ireland

^2^School of Biochemistry and Immunology, Trinity Biomedical Sciences Institute & Trinity College Institute of Neuroscience, Trinity College Dublin, Dublin 2, Rep. of Ireland

**Correspondence:**

John P. Lowry, Department of Chemistry, Maynooth University, Maynooth, Kildare, Rep. of Ireland.

Email: [John.Lowry@mu.ie](mailto:John.Lowry@mu.ie)

Phone: +353 (0)1 7084639


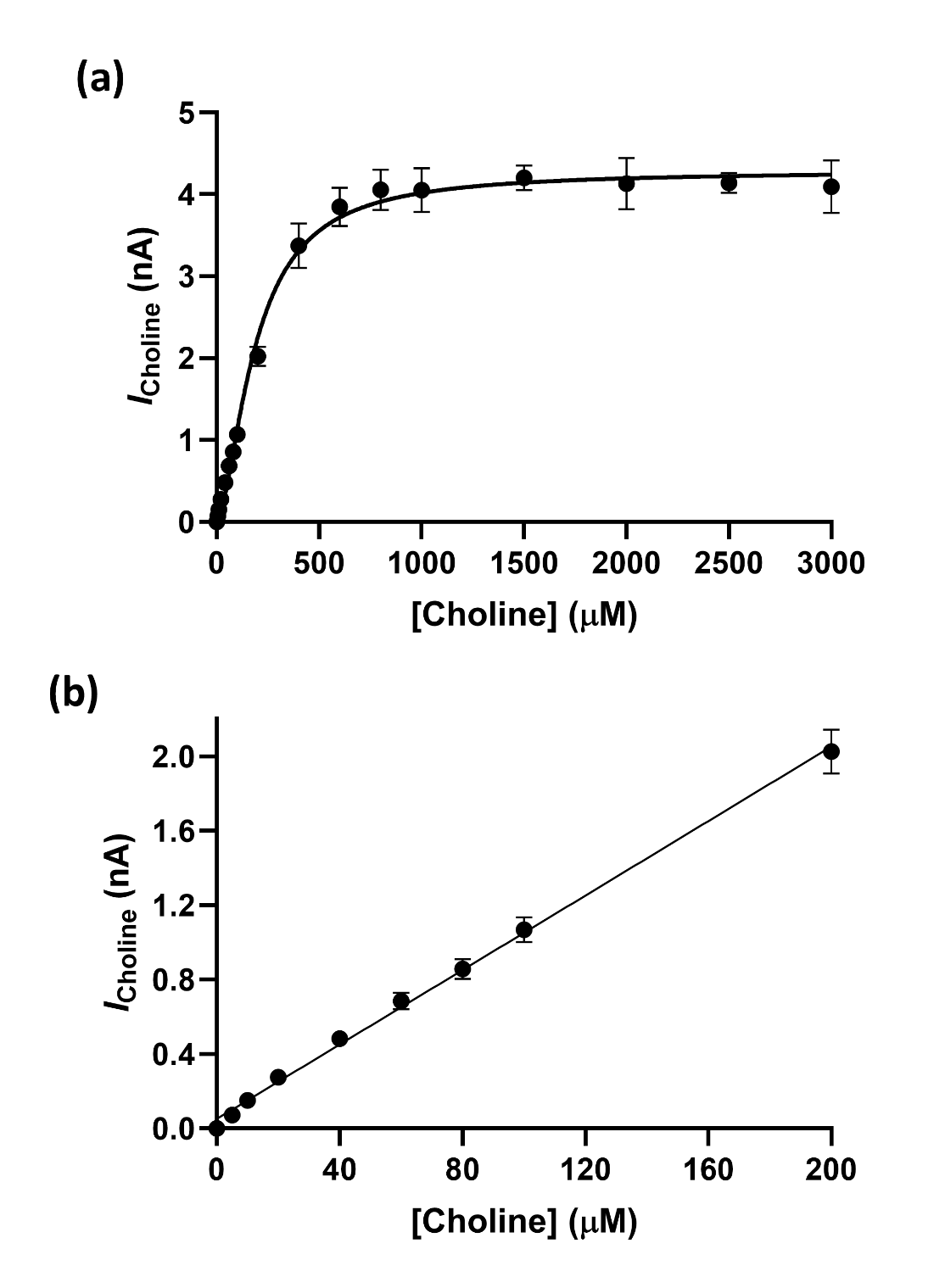


**SUPPLEMENTARY FIGURE S1: (a)** Choline calibration data (current *vs.* concentration) for Pt/PPD-PC:ChOx disc biosensors (*n* = 6) over the range 0 to 3,000 μM choline. **(b)** Linear regression analysis of choline concentrations ranging from 0 - 200 μM (sensitivity 2.26 ± 0.06 nA.mm^-2^.μM^-1^, *R^2^* = 0.962; limit of detection 0.14 ± 0.04 μM).


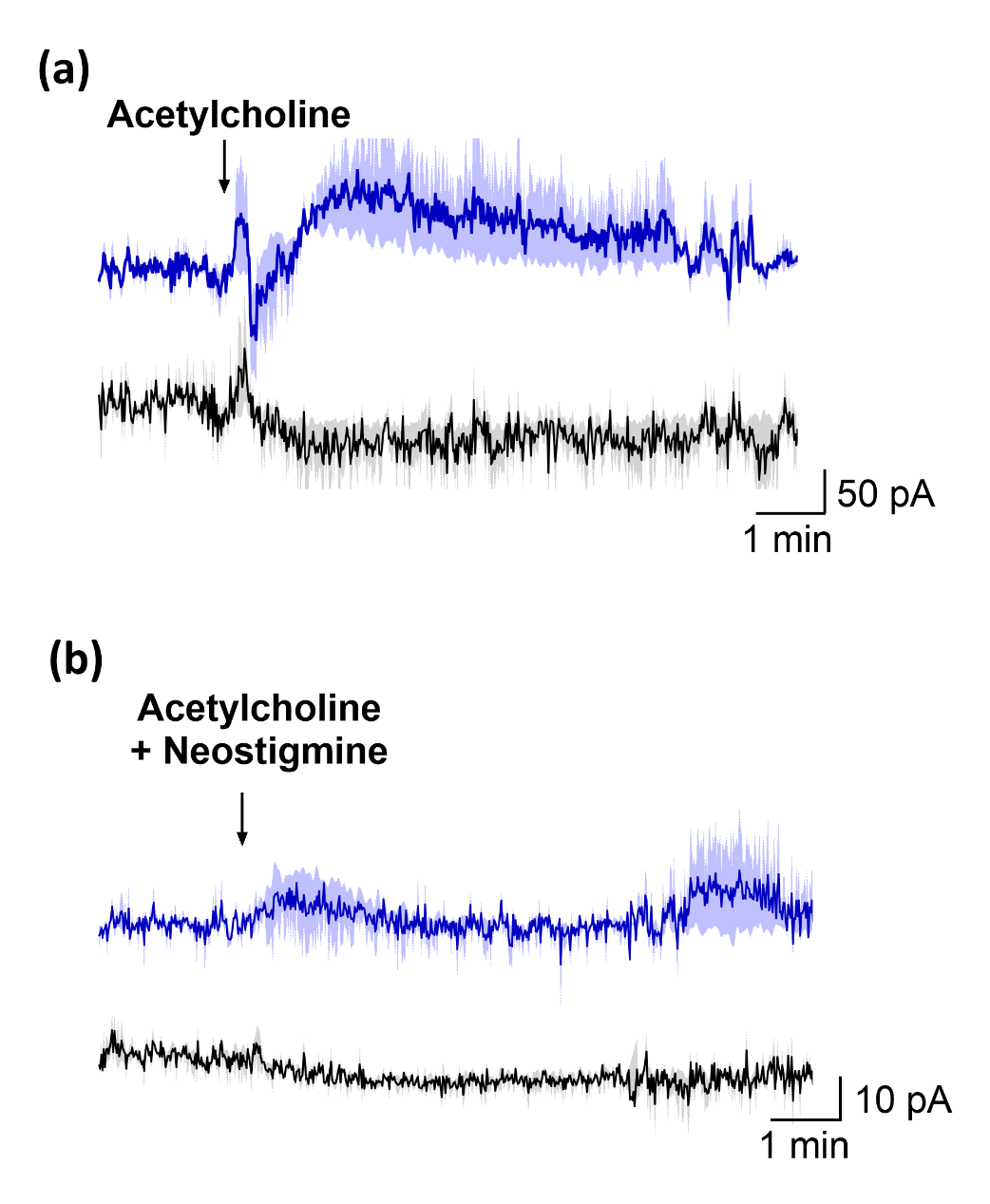


**SUPPLEMENTARY FIGURE S2:** Choline biosensor (blue) and null electrode (black) responses recorded in the hippocampus following the local pulse injection (800 nL at a rate of 80 nL/s) of acetylcholine (500 mM, **a**), and acetylcholine/neostigmine (500 mM/100 mM, **b**). Acetylcholine produced a maximum current increase of 90 ± 30 pA at 71 ± 6 s (*n* = 3). This was attenuated by the neostigmine with the current increasing by 3.4 ± 0.4 pA (*n* = 2) at the same time point following injection. No responses were observed at the null electrodes. Arrows indicate time point of injection and shadowing represents SEM.


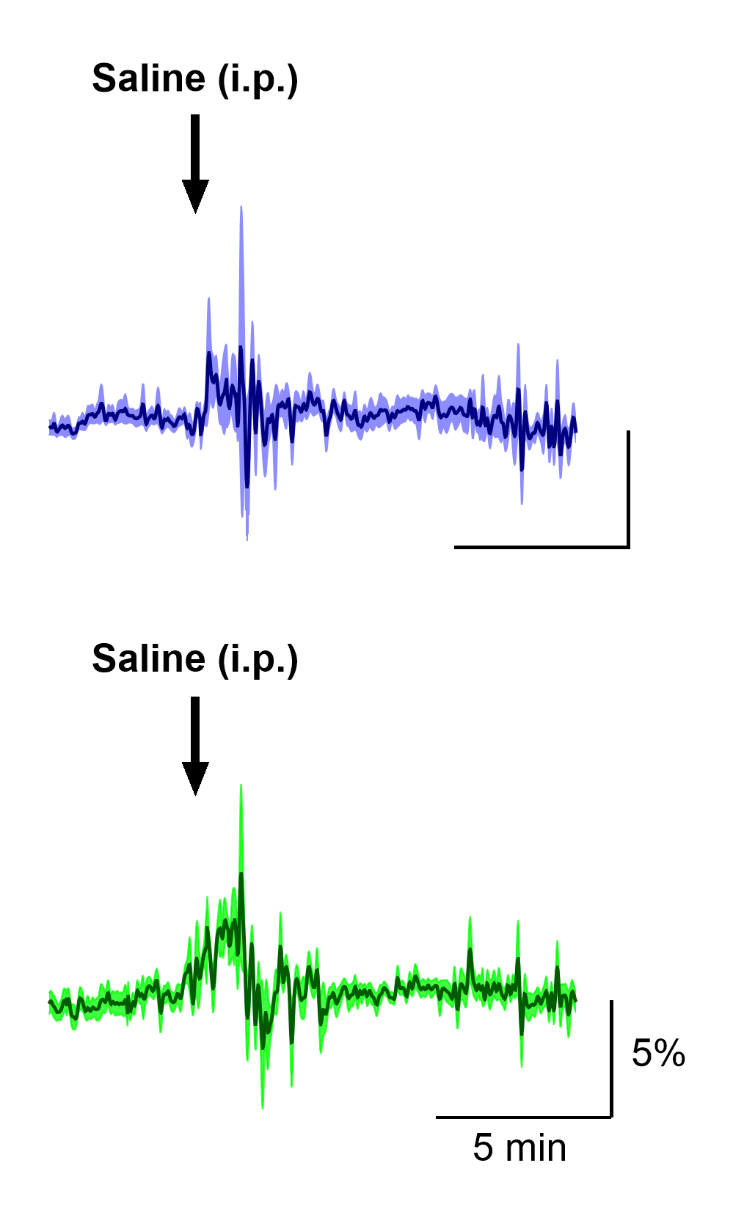


**SUPPLEMENTARY FIGURE S3:** Choline current responses (mean ± SEM) in the HPC (blue) and PFC (green) following an intraperitoneal injection of sterile saline (10 ml/kg). Data is normalised to the pre-injection baseline and presented as relative percentage current change over time. Arrows indicate time point of injection and shadowing represents SEM.

**
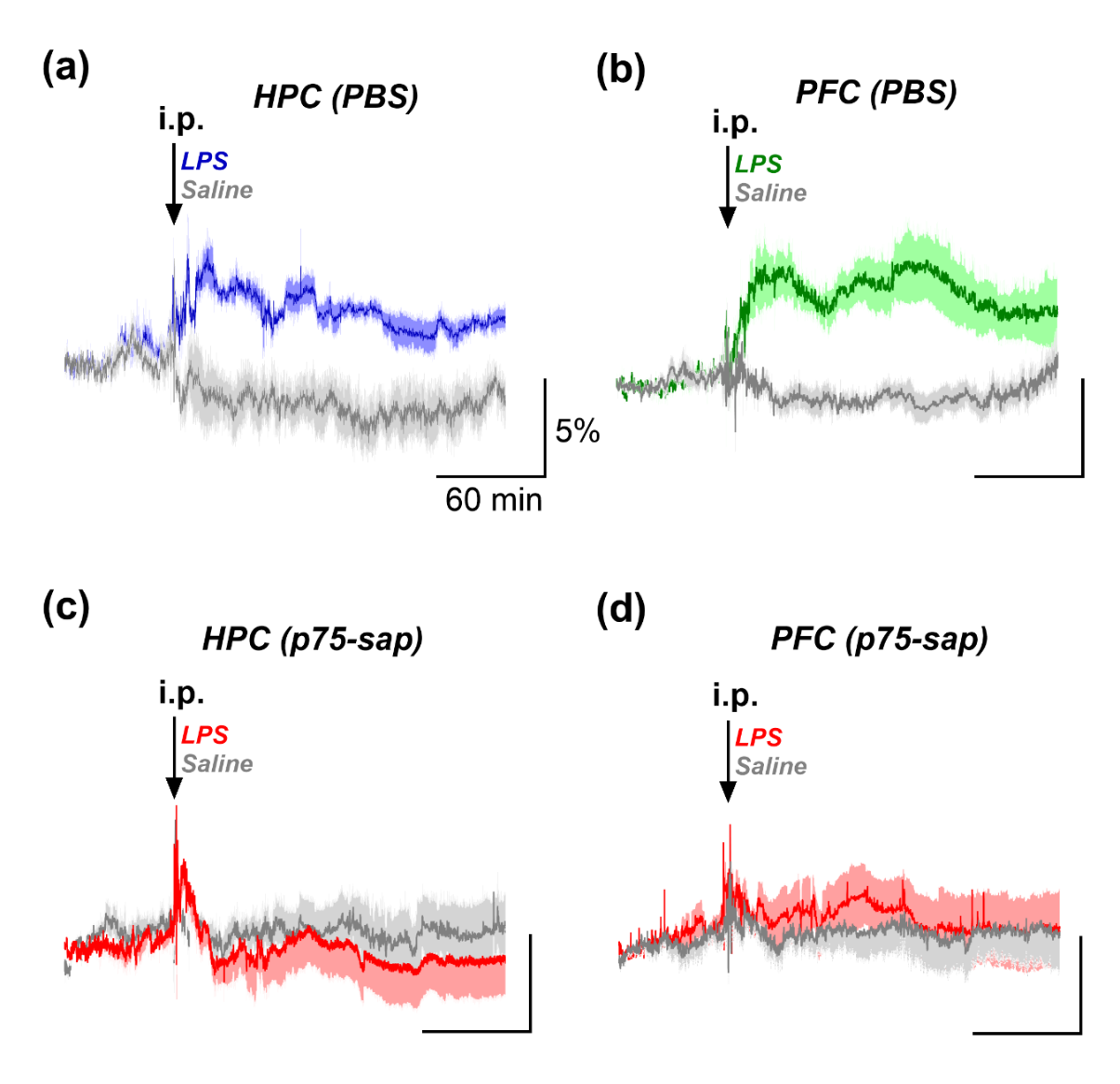
**

**SUPPLEMENTARY FIGURE S4:** Choline current responses (mean ± SEM) in the HPC and PFC from PBS control (**a**, **b**) and p75-sap (0.6 µg bilaterally) lesioned (**c**, **d**) mice following intraperitoneal injection (i.p.) of LPS (500 µg/kg; PBS – blue/green, p75 – red) or sterile saline (10 ml/kg, grey). Data is normalised to the pre-injection baseline and presented as relative percentage current change over time. Arrows indicate time point of injection and shadowing represents SEM.

In PBS control animals LPS produced a rapid increase in signal in both regions lasting several hours, which was significantly different (HPC *P* < 0.05, PFC *P* < 0.01; AUC, two-way ANOVA with Bonferroni *post-hoc* test) compared to saline only treatment. This LPS-induced increase was eliminated in both regions in the p75-sap lesioned animals (HPC & PFC *P* > 0.999), indicating the basal forebrain as the origin of the observed response. ***PBS control:*** HPC, **a** – LPS (blue, n=7), Saline (grey, n=6); PFC, **b** – LPS (green, n=10), Saline (grey, n=10). ***p75-sap:*** HPC, **c** – LPS (red, n=7), Saline (grey, n=7); PFC, **d** – LPS (red, n=6), Saline (grey, n=6).


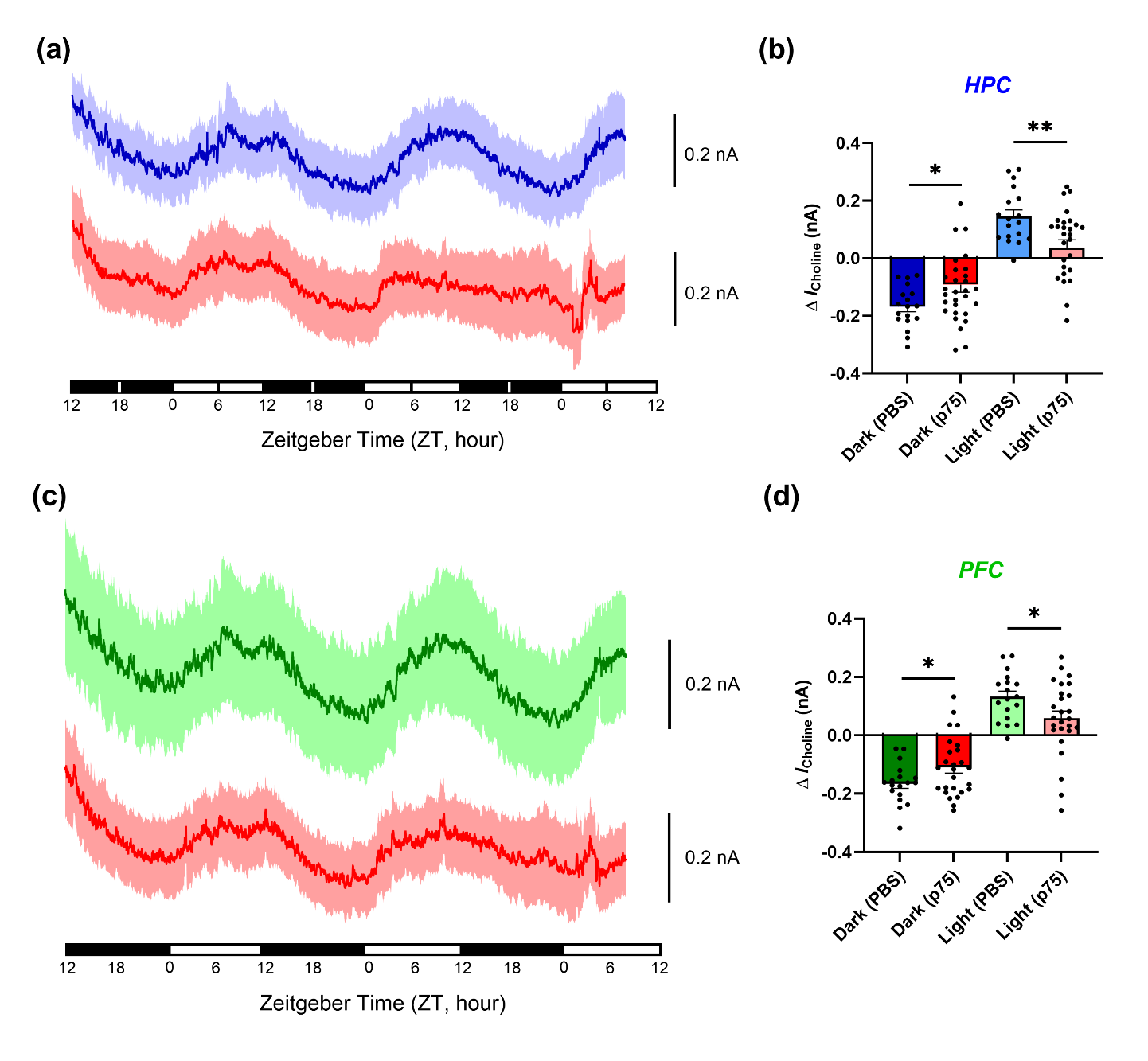


**SUPPLEMENTARY FIGURE S5:** Choline signal changes (mean ± SEM) in the HPC (**a**) and PFC (**c**) from PBS control (blue, green) and p75-sap lesioned (red) mice across three consecutive 12 hour light-dark phases. Lighting condition is indicated by means of Zeitgeber Time scale at the bottom of the figures; open bars (ZT 0-12 hours) indicate light phases, closed bars (ZT 12-0 hours) indicate dark phases.

Similar to untreated animals (Figure 4A), recorded choline current was significantly higher during the light phase in HPC and PFC regions in both the PBS control (HPC: *P* < 0.0001, *n* = 6; PFC: *P* < 0.0001, *n* = 6) and p75-sap (HPC: *P* < 0.01, *n* = 10; PFC: *P* < 0.0001, *n* = 9) animals. However, the current change was significantly reduced in the p75-sap animals in the light and dark phases in both regions (**b** and **d**): HPC (**b**) - Dark (PBS) *vs.* Dark (p75) * *P* < 0.05, Light (PBS) *vs.* Light (p75) ** *P <* 0.01; PFC (**d**) - Dark (PBS) *vs.* Dark (p75) * *P* < 0.05, Light (PBS) *vs.* Light (p75) * *P =* 0.05 (unpaired *t*-tests). Error bars represent SEM.
